# AI-powered detection of pumpkin leaf diseases using DualFusion-CBAM-stochastic for yield protection and precision agriculture

**DOI:** 10.3389/fpls.2025.1717226

**Published:** 2026-01-30

**Authors:** Ruchika Bhuria, Rahul Singh, Mudassir Khan, Mohamed Abbas, Jaibir Singh, Amel Ksibi, Nitin Kumar, Nitika Kapoor, Upinder Kaur

**Affiliations:** 1 Chitkara University Institute of Engineering and Technology, Chitkara University, Rajpura, Punjab, India; 2 Department of Computer Science, College of Computer Science, Applied College Tanumah, King Khalid University, Abha, Saudi Arabia; 3 Jadara Research Center, Jadara University, Irbid, Jordan; 4 Electrical Engineering Department, College of Engineering, King Khalid University, Abha, Saudi Arabia; 5 School of Computer Science & Engineering, Galgotias University, Greater Noida, Uttar Pradesh, India; 6 Department of Information Systems, College of Computer and Information Sciences, Princess Nourah bint Abdulrahman University, Riyadh, Saudi Arabia; 7 Department of Mechanical Engineering, Graphic Era (Deemed to be) University, Dehradun, Uttarakhand, India; 8 Department of Computer Science and Engineering (CSE), Chandigarh University, Mohali, Punjab, India; 9 School of Computer Science and Engineering, Lovely Professional University, Phagwara, Punjab, India

**Keywords:** computational plant pathology, Cucurbita pepo foliar disease, deep learning architecture, DualFusion-CBAM framework, image-based disease classification, precision agriculture, smart crop health monitoring

## Abstract

**Introduction:**

Early and accurate detection of pumpkin leaf diseases is essential for precision agriculture; however, manual inspection remains slow, subjective, and difficult to scale in real field environments. To address these limitations, this study proposes a robust deep-learning framework for automated pumpkin leaf disease classification.

**Methods:**

This study introduces DualFusion–CBAM–Stochastic, a hybrid deep-learning architecture that integrates two complementary convolutional backbones: DenseNet121 for fine-grained texture representation through dense connectivity and EfficientNetB3 for multi-scale contextual feature extraction using compound scaling. Input images are preprocessed through resizing to 224 × 224 pixels, ImageNet-based normalization, and controlled data augmentation, including horizontal and vertical flips, rotation, and zoom. Feature refinement is achieved using the Convolutional Block Attention Module (CBAM), which applies sequential channel and spatial attention, while stochastic-depth regularization improves generalization by randomly bypassing deep layers during training.

**Results:**

The proposed model was trained on a balanced dataset of 2,000 images across five pumpkin leaf disease categories. Experimental evaluation using ablation studies and comparative analysis against state-of-the-art models demonstrates that the proposed architecture achieves 96% classification accuracy, outperforming existing CNN-based approaches.

**Discussion:**

The results confirm that the synergistic integration of dual-backbone fusion, attention-guided refinement, and stochastic-depth regularization significantly enhances classification performance, feature interpretability, and model stability under diverse visual conditions. These findings advance automated pumpkin leaf disease diagnosis and provide a strong methodological foundation for future research in agricultural image analysis.

## 1 Introduction

Cucurbita pepo (pumpkin) is a high-value horticultural crop widely cultivated across diverse agro-climatic regions, yet it remains highly vulnerable to multiple foliar diseases that adversely affect physiological growth, leaf function, and overall yield stability (Chen et al., 2025a). Early and reliable disease identification is therefore essential for enabling timely intervention, sustaining crop productivity, and reducing economic losses. Among the major diseases affecting pumpkin foliage—such as Powdery Mildew, Downy Mildew, Bacterial Wilt, and Anthracnose—fungal and bacterial pathogens induce a spectrum of symptoms including leaf chlorosis, necrotic lesions, wilting, and premature senescence (Chen et al., 2025b). These infections spread rapidly under favorable environmental conditions, causing significant field-level damage if not detected and managed promptly (Jiang et al., 2022; Sethy et al., 2019). Recent studies in plant disease surveillance and agricultural informatics highlight a growing need for scalable, accurate, and automated diagnostic systems capable of supporting modern precision-agriculture practices (Bagga and Goyal, 2024; Jafar et al., 2024; Omaye et al., 2024; Wang et al., 2025; Wang et al., 2024).

Conventional disease diagnosis relies primarily on visual inspection by farmers, agronomists, or plant-health specialists. Although expert evaluation can be reliable, this manual approach is inherently subjective, labor-intensive, and often infeasible in large-scale production environments. Limited availability of trained personnel, the presence of diseases with overlapping visual symptoms, and the complexity of field conditions frequently lead to misdiagnosis or delayed treatment. These challenges are particularly acute in remote or resource-constrained agricultural regions, where dependence on manual disease inspection contributes to substantial yield losses and inefficient crop-management decisions (Mithu et al., 2022; Paymode and Malode, 2022).

Advances in artificial intelligence and computer vision have accelerated the adoption of deep-learning approaches for automated crop-disease recognition. Convolutional Neural Networks (CNNs), in particular, have demonstrated strong performance in identifying plant diseases from leaf images and have been incorporated into IoT-enabled and mobile-based agricultural monitoring platforms (Ngugi et al., 2024). Despite this progress, most existing CNN architectures exhibit limited generalization under real-world conditions. Images captured in natural field environments are affected by varying illumination, shadows, leaf occlusion, heterogeneous backgrounds, and irregular lesion morphology, all of which introduce noise and degrade classification accuracy. Moreover, conventional single-backbone CNNs often extract redundant or shallow representations, which restricts their capacity to differentiate visually similar disease patterns. Consequently, many previously reported models perform well on controlled datasets but struggle to maintain robustness when exposed to diverse agricultural imagery (Chen et al., 2025b; Liu et al., 2025). These limitations underscore the necessity for more adaptive and feature-rich architectures capable of learning both localized lesion textures and broader contextual cues.

To address these field-level challenges, it is essential to utilize a model capable of capturing both fine-grained lesion details and broader contextual structures. Single-backbone CNNs often struggle in such conditions because they extract either highly localized or overly generalized features, which limits their ability to differentiate visually similar symptoms under diverse illumination and background variations. Therefore, a dual-path architecture becomes necessary. DenseNet121 contributes dense local texture representation suitable for capturing subtle lesion patterns, while EfficientNetB3 offers multi-scale contextual learning optimized through compound scaling. Integrating these two streams allows the model to learn richer and more discriminative features. To further refine the extracted representations, the Convolutional Block Attention Module (CBAM) emphasizes disease-relevant spatial and channel-wise information and suppresses irrelevant background noise. Additionally, stochastic-depth regularization improves robustness by reducing overfitting, which is common in agricultural datasets with heterogeneous image conditions. These motivations collectively justify the introduction of the DualFusion-CBAM-Stochastic model for robust and interpretable pumpkin leaf-disease classification.

To overcome these challenges, this study proposes DualFusion-CBAM-Stochastic, a hybrid deep-learning architecture specifically tailored for robust pumpkin leaf-disease classification under heterogeneous field conditions. The framework integrates two complementary convolutional backbones—DenseNet121 for detailed local feature extraction through dense connectivity and EfficientNetB3 for multi-scale contextual learning via compound scaling. This dual-path design enhances feature diversity and facilitates more discriminative representation learning. The model further incorporates the Convolutional Block Attention Module (CBAM) to emphasize disease-relevant spatial and channel-wise features while suppressing background interference, thereby improving interpretability and predictive reliability. Additionally, stochastic-depth regularization is employed to mitigate overfitting and enhance model generalization by randomly bypassing selected deep layers during training. This synergistic integration enables the architecture to more effectively handle complex agricultural image characteristics, outperform traditional CNNs, and maintain stable performance across diverse disease categories (Wang H. et al., 2024).

The primary contributions of this study are fourfold: (i) the development of a novel hybrid dual-backbone architecture designed specifically for pumpkin leaf-disease recognition; (ii) the integration of attention-guided refinement and stochastic-depth regularization to enhance robustness and interpretability; (iii) a comprehensive evaluation involving ablation analyses and comparisons with contemporary CNN and transformer-based models; and (iv) an assessment of the model’s suitability for practical agricultural deployment. By addressing the inherent challenges of real-world field imagery, this work advances automated plant-disease detection and contributes to the broader goal of intelligent, sustainable, and data-driven crop-management systems.

- A novel dual-branch model is proposed by fusing two powerful convolutional fine-tuned neural networks, DenseNet121 and EfficientNetB3, to extract diverse and complementary feature representations. This fusion enhances the discriminative ability of the model in classifying complex patterns of pumpkin leaf diseases.
- To improve feature refinement, the CBAM is included after the global-average-pooling layers in both branches of the network. This attention mechanism enables the model to adaptively focus on the most relevant spatial and channel-wise features, improving classification accuracy.
- To mitigate overfitting and enhance generalization, stochastic depth is employed in both branches. By randomly skipping layers during training, the model benefits from implicit ensemble behavior and improved robustness.

The proposed DualFusion-CBAM-Stochastic framework represents a significant advancement in developing automated, reliable, and interpretable plant-disease-detection systems that can increase agricultural productivity and reduce economic losses. The findings of this research provide valuable insights into the potential of AI-based methods for sustainable farming and plant-disease diagnosis. The structure of the paper is as follows: Section 2 covers related work, summarizing previous research in plant disease detection using CNN architectures and identifying existing limitations. Section 3 explains the proposed methodology, including the fusion of DenseNet121 and EfficientNetB3 models, the use of Swish activation, and regularization techniques for enhanced classification. The Results and Discussion section evaluates model performance using accuracy, precision, recall, F1-score, and confusion-matrix analysis. Section 5 presents the ablation study, highlighting the individual contributions of CBAM and stochastic depth by comparing different model variants. Section 6 discusses the state-of-the-art comparison, benchmarking the proposed model against existing approaches to demonstrate its superior performance. Finally, Section 7 delivers the conclusion and future work, summarizing the study’s findings and proposing future directions such as real-time deployment, model optimization, and extension to other plant-disease-classification tasks.

## 2 Related work

The rapid advancement of artificial intelligence (AI) and deep learning has significantly transformed agricultural diagnostics, particularly for pumpkin leaf disease detection. Early studies such as Khandaker et al. (2024) introduced an explainable AI–enhanced framework for pumpkin leaf disease classification using multiple CNN architectures, where ResNet50 achieved the highest accuracy through Grad-CAM and Score-CAM visualization. Their work demonstrated the importance of interpretability but did not address field-level generalization challenges. Tian et al. (2025) explored pumpkin leaf physiology through Structural Equation Modeling, highlighting key agronomic indicators but not focusing on image-based disease recognition. Singh and Kumar (2024) developed a Constitutive Artificial Neural Network (CANN) incorporating Gabor filters for classifying Powdery Mildew, White Blight, and Downy Mildew, showing strong performance on controlled datasets but with limited adaptability to real-field variability. Similarly, Prasad et al. (2024) designed a YOLOv8l-based system for red beetle detection in pumpkin fields, demonstrating real-time pest monitoring, though their approach targeted pest identification rather than foliar disease classification. Supakitthanakorn et al. (2024) proposed a multiplex PCR assay to detect mixed begomovirus infections in pumpkin leaves, contributing valuable molecular-level insights but not providing deep-learning-based solutions.

Several non-pumpkin studies further demonstrate the evolution of deep learning in agricultural imaging and highlight gaps addressed in this study. Hoque et al. (2025) compared ResNet50 and MobileNet for tomato leaf disease classification, confirming the importance of residual learning for feature extraction. Bezabh et al. (2024) proposed ResLeNet, a hybrid CNN combining ResNet and LeNet, achieving high accuracy but relying on single-backbone structures. Haider et al. (2024) introduced RBNet-Self using residual blocks and self-attention for cotton and wheat leaves, achieving strong accuracy yet without addressing dual-path feature extraction. Li et al. (2024) and Tej et al. (2024) improved YOLOv5-based models for leaf disease detection and real-time localization, highlighting the value of lightweight architectures for deployment but lacking attention-driven multi-scale fusion. Transformer-based architectures have also been explored, such as SEViT by Zeng et al (Zeng et al., 2023), which combined SE attention with vision transformers, achieving competitive accuracy but requiring large datasets and high computational cost.

Traditional machine-learning methods also contributed valuable foundations. Lin et al. (2021) employed PCA and SVM for pumpkin powdery mildew classification, achieving strong accuracy through handcrafted features. Yuan et al. (2021) developed SPEDCCNN for disease segmentation with high Dice scores, focusing on encoder-decoder structures rather than multi-backbone fusion. Bozkurt (2020) utilized UAV imagery and Random Forest classification to map pumpkins and flowers, demonstrating scalable monitoring but not disease-focused analysis. Argüeso et al. (2020) addressed limited labeled data through Few-Shot Learning using InceptionV3, highlighting the effectiveness of transfer learning in low-data environments. Furthermore, attention mechanisms and stochastic-depth regularization have been shown to significantly enhance feature representation and model generalization in CNN-based architectures. The Convolutional Block Attention Module (CBAM) (Woo et al., 2018) adaptively refines channel-wise and spatial features, enabling the model to focus on the most informative regions of an image. Similarly, stochastic-depth regularization (Huang et al., 2016) introduces layer-wise random dropping during training, which mitigates overfitting and improves robustness under heterogeneous data conditions. Integrating these techniques into dual-backbone CNN frameworks allows for improved discrimination of subtle disease patterns in complex agricultural imagery, addressing limitations observed in prior single-backbone and attention-limited models.

Overall, existing studies demonstrate substantial progress but also reveal critical gaps: (i) limited robustness under heterogeneous field conditions, (ii) reliance on single-backbone CNNs, (iii) insufficient integration of attention mechanisms with multi-scale feature extraction, and (iv) limited interpretability in disease classification. The proposed DualFusion-CBAM-Stochastic framework addresses these gaps by combining dual-path convolutional backbones, attention-guided refinement, and stochastic-depth regularization, providing a more robust and generalizable solution for pumpkin leaf disease classification. **Table 1** shows the comparative summary of existing studies on plant disease detection based on dataset, technique, accuracy, and key findings.

**Table 1:** Comparative summary of existing studies on plant disease detection based on dataset, technique, accuracy, and key findings.

Ref no & year	Dataset used	Technique name	Accuracy	Key findings
(Khandaker et al., 2024) /2025	Pumpkin Leaf Disease Dataset	ResNet50 (Explainable AI)	90.50%	ResNet50 outperformed other CNN architectures in detecting pumpkin leaf diseases, achieving the highest accuracy. Explainable AI techniques like Grad-CAM enhanced model interpretability.
(Tian et al., 2025) /2025	Pumpkin Leaf Growth Dataset	Structural Equation Modeling (SEM)	93.90%	Utilised the SEM to look at how different amounts of water and fertiliser affected the growth of pumpkin leaves, photosynthesis, and yield.
(Hoque et al., 2025) /2025	Tomato Leaf Disease	ResNet50	92.53%	ResNet50 outperformed other CNNs in detecting pumpkin leaf diseases, with Grad-CAM enhancing model interpretability.
(Bezabh et al., 2024) /2024	Hybrid Dataset for Pumpkin Disease	Hybrid Classification Approach	90.50%	Proposed a hybrid approach combining multiple classifiers to improve pumpkin disease classification.
(Singh and Kumar, 2024) /2024	Plant Leaf Disease Dataset	Constitutive Artificial Neural Network (ANN) with Gabor Filters	97.56 %	Utilized Gabor filters with ANN to enhance plant leaf disease detection.
(Prasad et al., 2024) /2024	Agricultural Field Crop Dataset	Deep Learning Multiplex PCR	89.00%	Developed a smart pest detection system for crops using deep learning techniques.
(Supakitthanakorn et al., 2024) /2024	Pumpkin Virus Dataset	Multiplex Polymerase Chain Reaction (PCR)	98.50%	Detected mixed infections of three begomoviruses in pumpkin using multiplex PCR.
(Haider et al., 2024) /2024	Tomato Leaves dataset	LSGNet (Lightweight Sandglass Network)	95.54%	LSGNet achieved the highest accuracy of 95.54% with only 0.75 million parameters, making it the most efficient and accurate model for tomato disease recognition.
(Li et al., 2024) /2024	Leaf Disease Dataset	Improved YOLOv5 Deep Learning Model	92.40 %	Introduced a lightweight algorithm based on an improved YOLOv5 model for recognizing pear leaf diseases in natural scenes.
(Tej et al., 2024) /2024	Leaf Disease Dataset	Deep Learning for identifying and Identifying Diseases	94.00%	Developed an affordable deep learning-based system for leaf disease detection and localization to aid precision agriculture.
(Zeng et al., 2023)/ 2023	Large-Scale Plant leaves Disease Dataset	SEViT (Transformer & Attention Convolution)	81.00%	Proposed SEViT, a model combining transformers and attention convolution for large-scale, fine-grained plant disease classification.
(Lin et al., 2021) /2021	Pumpkin Powdery Mildew Dataset	PCA and machine learning for image processing	97.30 %	Identified pumpkin powdery mildew using image processing techniques combined with PCA and machine learning.
(Yuan et al., 2021) 2021	Crop Disease Leaf Dataset	SPEDCCNN (Encoder-Decoder CNN)	90.00%	Introduced SPEDCCNN, a cascade of encoders and decoders CNN for separating leaves with agricultural disease.
(Bozkurt, 2020) /2020	UAV Imagery Dataset	Object-Based Classification (SEViT)	88.34 %	Used UAV images to classify pumpkins and pumpkin flowers by looking at the objects in the images.
(Argüeso et al., 2020) /2020	Field Images Dataset	(InceptionV3) Few-Shot Learning for Classifying Plant Diseases	94.00 %	Used a few-shot learning method called InceptionV3 to classify plant diseases using pictures taken in the field.

## 3 Proposed methodology

The initial phase of the proposed pumpkin leaf disease detection framework (**Figure 2**) involves data preprocessing, where all input images are resized to 224×224 pixels and divided into training, validation, and testing subsets using an 80:10:10 ratio. To enhance model generalization and mitigate overfitting, several data augmentation techniques, including rotation, shifting, and zooming, are applied. Following augmentation, the images are normalized to scale pixel values between 0 and 1, ensuring uniform intensity distribution across the dataset and stable model convergence.

**Figure 1:**
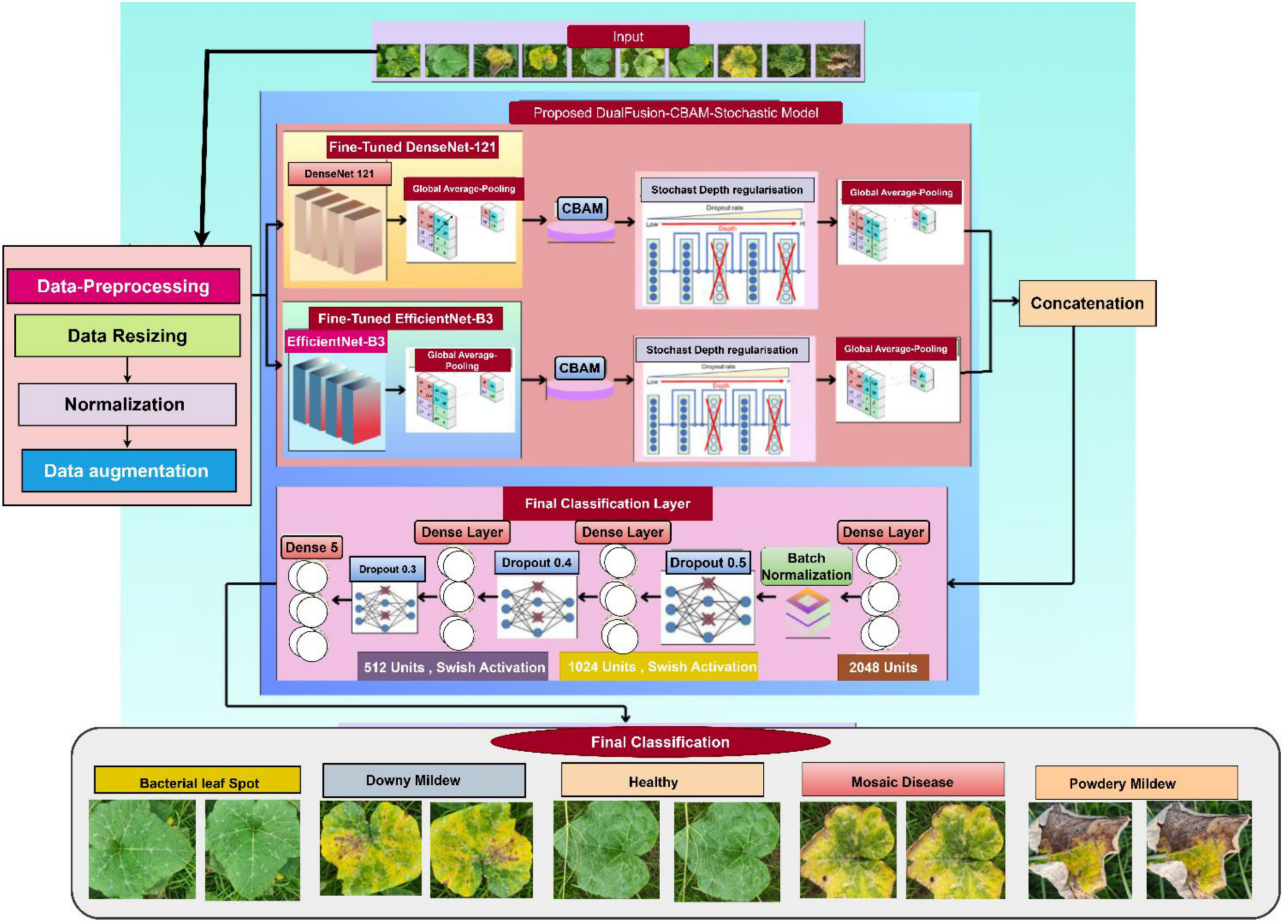
Proposed methodology for pumpkin leaf disease classification using dual feature extraction, CBAM attention, and feature fusion.

As illustrated in **Figure 1**, the proposed DualFusion-CBAM-Stochastic model integrates dual feature extraction, attention-based refinement, and multi-level feature fusion for robust pumpkin leaf disease classification. DenseNet121 and EfficientNetB3 serve as the two primary backbone architectures for feature extraction. These pre-trained CNNs utilize multiple convolutional layers to capture low-, mid-, and high-level features from input images, providing a strong foundation for visual representation and disease identification. The fusion of DenseNet121 and EfficientNetB3 was deliberately chosen due to their complementary strengths. DenseNet121 promotes feature reuse and efficient gradient propagation through dense connectivity, effectively capturing fine-grained textural patterns of diseased leaf regions. EfficientNetB3, on the other hand, employs compound scaling to balance network depth, width, and resolution, enabling efficient extraction of global contextual features with reduced computational cost. By combining these two backbones, the proposed model overcomes the limitations of single CNN architectures, which often exhibit feature redundancy and limited adaptability to complex lighting and texture variations. The integration of the Convolutional Block Attention Module (CBAM) allows the network to adaptively emphasize disease-relevant regions through channel and spatial attention mechanisms, while stochastic-depth regularization enhances model robustness by randomly dropping residual layers during training. Specifically, the Convolutional Block Attention Module (CBAM) is deployed in a hybrid configuration that sequentially integrates channel and spatial attention mechanisms to enhance representational quality. The channel attention sub-module adaptively recalibrates feature responses by leveraging both global average and max pooling operations, followed by a shared multi-layer perceptron (MLP) to capture inter-channel dependencies and emphasize the most informative feature maps. Subsequently, the spatial attention sub-module applies a convolutional transformation over the aggregated channel descriptors to localize disease-specific spatial patterns within the leaf images. This two-stage attention mechanism enables the model to simultaneously discern “what” features are most critical and “where” they are spatially distributed, thereby substantially improving its discriminative strength, robustness, and interpretability. Stochastic depth regularization is selectively applied to the deeper convolutional layers of both DenseNet121 and EfficientNetB3 branches to mitigate overfitting and enhance generalization. A linearly decaying survival probability schedule is adopted, where shallow layers maintain a high survival probability (approximately 1.0), gradually decreasing to 0.8 in deeper layers. This selective dropout of residual layers encourages implicit model ensembling, improving robustness without sacrificing convergence stability. By applying stochastic depth primarily to deeper layers, the framework preserves essential low-level features while promoting diversity in higher-level feature representations. Together, these enhancements make the DualFusion-CBAM-Stochastic framework more discriminative, interpretable, and generalizable than conventional single-backbone CNN models. The refined feature maps generated through the dual backbone and attention modules are subsequently passed through a global average pooling layer and concatenated to form a unified feature vector. Before concatenation, the feature maps generated by DenseNet121 and EfficientNetB3 are aligned to ensure dimensional compatibility. Since each backbone produces features of different spatial and channel resolutions, a Global Average Pooling (GAP) layer is applied at the end of both branches to convert the spatial feature maps into fixed-length vectors. When minor channel mismatches occur, a 1×1 convolutional projection layer is used to normalize the number of channels. This alignment guarantees smooth concatenation along the feature dimension, preserving semantic integrity and ensuring that both fine-grained and contextual information are equally represented in the fused feature vector. This composite feature representation is processed through a series of fully connected layers comprising 2048, 1024, and 512 neurons, each followed by batch normalization, Swish activation, and dropout regularization to prevent overfitting. The final dense layer employs a sigmoid activation function for binary classification and a softmax activation function for multi-class scenarios.The model is trained using the AdamW optimizer, which incorporates weight decay regularization to ensure stable and efficient convergence. Additionally, a categorical cross-entropy loss function with label smoothing is employed to promote smoother decision boundaries and reduce overfitting. To address dataset imbalance, class weighting is applied during training, ensuring fair contribution from each class. Overall, the proposed DualFusion-CBAM-Stochastic framework combines dual-path feature extraction, attention-guided refinement, and stochastic-depth regularization to achieve superior accuracy, robustness, and interpretability in pumpkin leaf disease classification.

**Figure 2:**
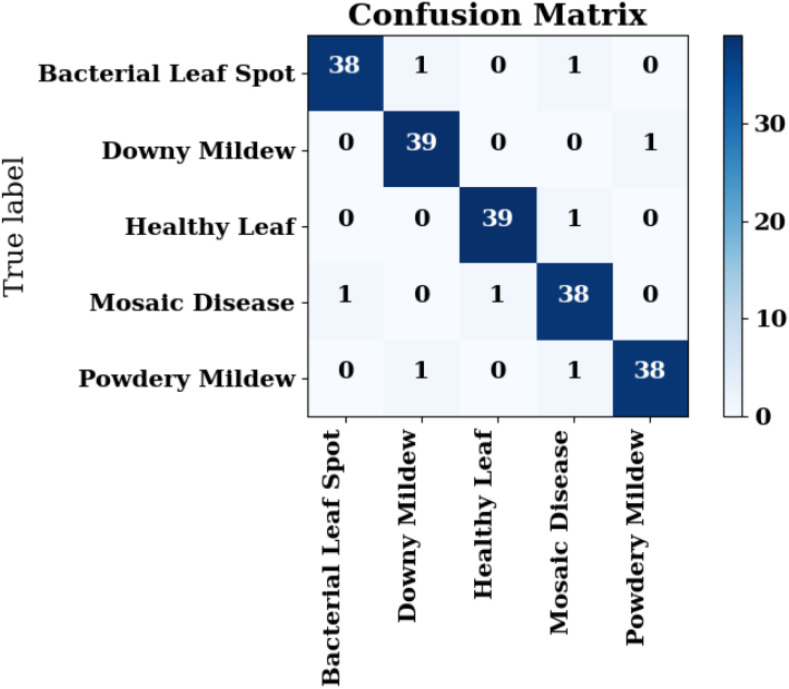
Confusion matrix of the DualFusion-CBAM-Stochastic model.

### 3.1 Input dataset

**Figure 3:**
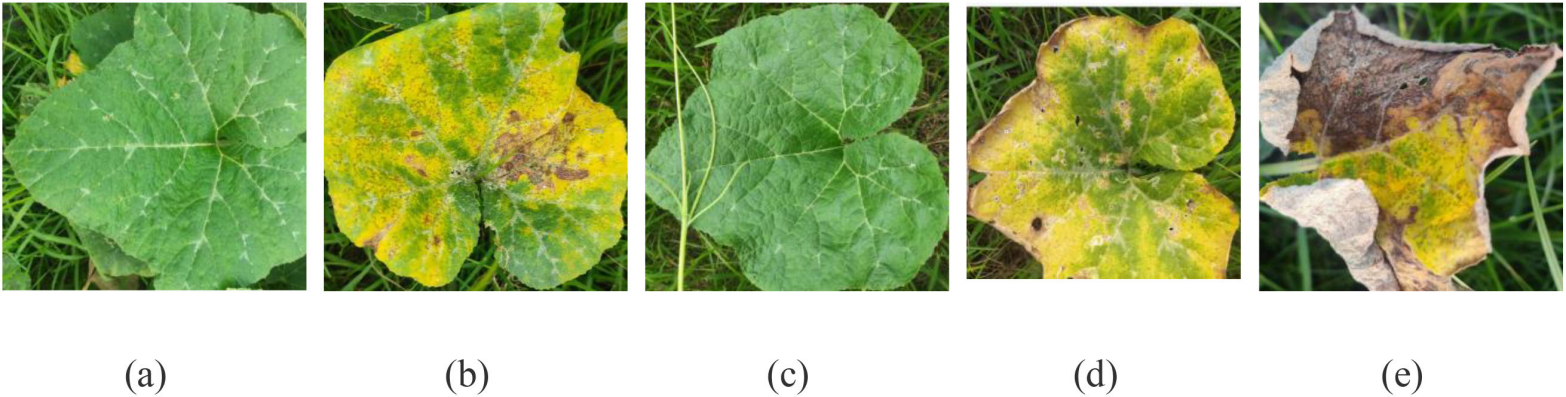
Pumpkin leaves dataset **(a)** Bacterial Leaf Spot, **(b)** Downy Mildew, **(c)** Healthy Leaf, **(d)** Mosaic Disease, **(e)** Powdery Mildew.

The Pumpkin Leaf Disease Dataset comprises 2, 000 high-resolution images of pumpkin leaves, systematically collected from agricultural regions in Bangladesh. The dataset is organized into five categories, namely Bacterial Leaf Spot, Downy Mildew, Healthy Leaf, Mosaic Disease, and Powdery Mildew, and Healthy leaves, providing a comprehensive representation of common foliar conditions. All classes are stored in separate folders, and it is easy to traverse and efficiently preprocess the data for machine learning. All images are provided in plain JPEG format, and thus they can readily be supported by most image processing packages and deep learning platforms. The data aims at aiding many types of research and application uses such as plant disease diagnosis using automation system, analysis of symptoms, and agricultural science teaching materials. High resolution images ensure explicit display of the visible symptoms like discoloration, texture modification, and spots as against features and can prove valuable in precision categorization. To develop and evaluate the model, the dataset is partitioned into training, validation, and testing sets using an 80:10:10 split, resulting in 1, 600 images for training, 200 for validation, and 200 for testing. A stratified sampling approach is applied to maintain class distribution across all subsets, ensuring that each disease type and healthy class is proportionally represented. The training set is utilized for model learning, while the validation set is employed for hyperparameter tuning and interim performance assessment. The test set is reserved for evaluating the model’s generalization ability. This systematic data partitioning facilitates the development of robust and accurate deep learning models for the classification of pumpkin leaf diseases. The dataset utilized in this study comprises 2, 000 high-resolution RGB images of Cucurbita pepo (pumpkin) leaves, systematically categorized into five distinct disease classes: Bacterial Leaf Spot, Downy Mildew, Healthy Leaf, Mosaic Disease, and Powdery Mildew. The images were primarily obtained from theKaggle public repository [https://www.kaggle.com/datasets/muhammadardiputra/pumpkin-leaf-disease-dataset ] and were further supplemented with field-captured samples to enhance diversity in terms of lighting conditions, leaf orientation, and background texture. Each image was resized to 224 × 224 pixels and normalized within a [0, 1] range to ensure consistency across the dataset. All images were resized to 224 × 224 pixels and normalized using the standard ImageNet statistics (mean = [0.485, 0.456, 0.406], std = [0.229, 0.224, 0.225]) to ensure compatibility with the pretrained DenseNet121 and EfficientNetB3 models. To improve model generalization and mitigate potential class imbalance, data augmentation techniques such as random rotation, flipping, zooming, and brightness adjustments were applied, expanding the training set to approximately 4, 800 images. The dataset was then partitioned into training (80%), validation (10%), and testing (10%) subsets to ensure robust and unbiased model evaluation. As depicted in **Figure 3** the input dataset comprises five distinct classes with an equal number of images, ensuring balanced representation across categories.

### 3.2 Data preprocessing

**Figure 4:**
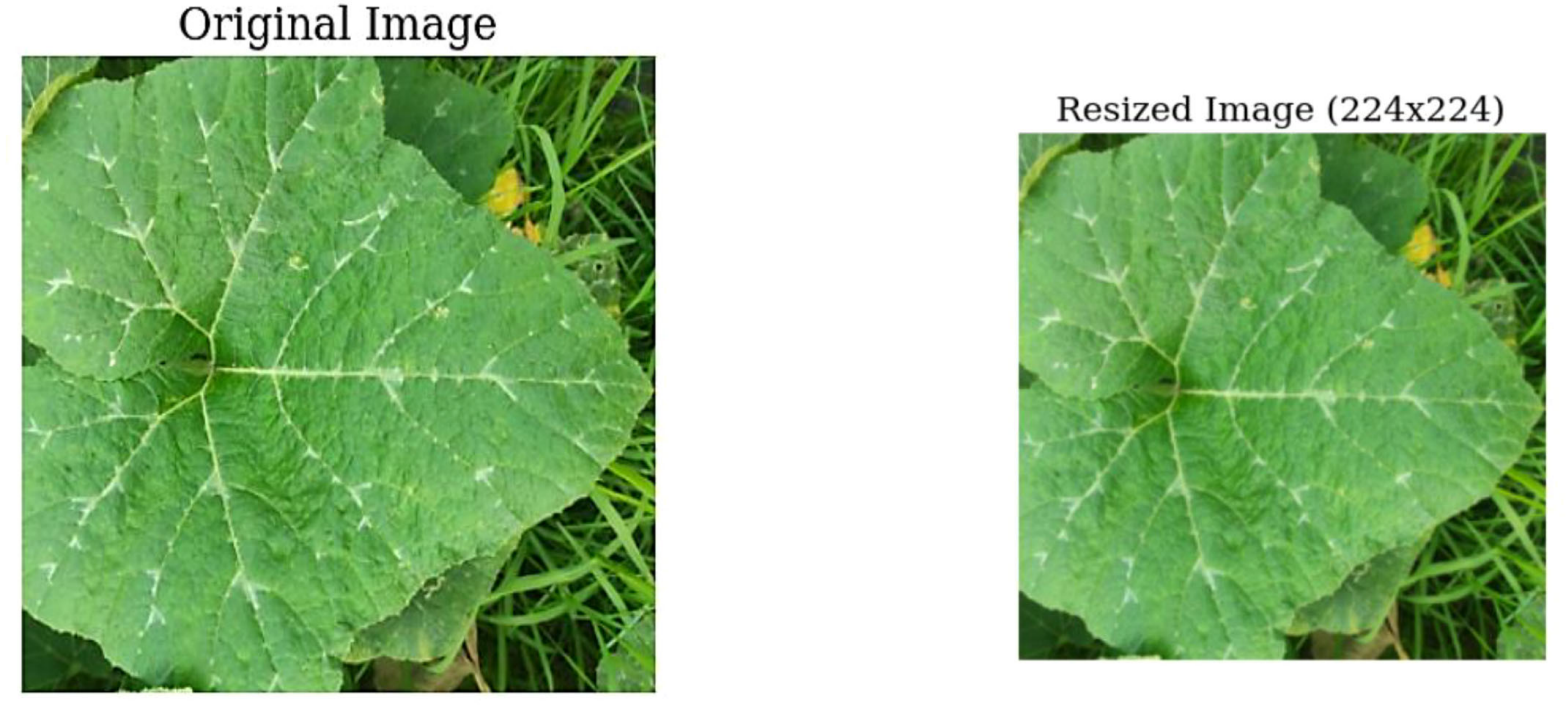
Data resizing step converting original images to fixed dimensions, facilitating structured input to DenseNet121 and EfficientNetB3.

Effective preprocessing of input data constitutes a fundamental step in the development of deep learning models, significantly influencing their accuracy, reliability, and overall performance. For the Pumpkin Leaf Disease dataset, several preprocessing techniques were applied to enhance model performance, ensure consistency, and improve generalization. Proper use of data preprocessing is prerequisite to the good performance by the model and the sound quality of classification results. This paper analyzed Pumpkin Leaf Disease dataset using an extensive preprocessing pipeline to normalize data, enhance training and avoid overfitting. All images within the dataset were uniformly resized to 224 × 224 pixels, ensuring consistency in input dimensions and compatibility with standard deep learning architectures during both training and evaluation phases. All the images will be resized into a predetermined resolution of 224 x 224 pixels, so they may be used with pre-trained deep learning models, DenseNet121 and EfficientNetB3. Such standardization of input dimensions allows efficient batch training and maintains necessary visual features of the symptoms of a disease. [0, 1] by applying min-max scaling. This activity scales input data distribution to meet the expectations of the pre-trained model, which results in more rapid convergence, besides enhanced stability of training. To ensure further robustness of the model data augmentation methods were used on the training data. These were flips and rotations in all the four directions (horizontally, vertically, randomly at +/-30 degrees) and zooming at +/-20 percent. All transformations mimic the leaf appearing as encountered in a real-world, with variation in leaf orientation, scale and perspective, prompting the model to learn to be invariant to these aspects. A combination of these preprocessing procedures made the input pipeline of high quality that can lead to proper and generalizable disease classification. The resizing of all input images to a fixed resolution of 224×224 pixels is illustrated in **Figure 4**.

#### 3.2.1 Data augmentation

Data augmentation serves as a crucial preprocessing step to increase the diversity of the training dataset and enhance the generalization capability of deep learning models. In this study, four augmentation techniques horizontal flipping, vertical flipping, rotation, and zooming were applied to the pumpkin leaf images to artificially expand the dataset and simulate realistic variations encountered in agricultural field environments. These geometric transformations mimic changes in leaf orientation, camera angle, and scale, enabling the model to learn feature invariances and reducing the risk of overfitting. Importantly, augmentation was applied only to the training set, while validation and test sets were kept unchanged to ensure unbiased model evaluation. The following augmentation operations were utilized:

This operation reflects the image across its vertical axis, as represented mathematically in Equation 1. These augmentation techniques enhance the model’s capability to detect leaf characteristics that can occur in symmetrical patterns in real-world environments.

(1) $$I_{hflip}(x,\;y)=I(W-x-1,y)$$

Vertical flipping inverts the image along the horizontal axis and is mathematically given in Equation 2. This operation accounts for leaf images captured from upside-down perspectives.

(2) $$I_{hflip}(x,y)=1(x,W-y-1)$$

To incorporate angular invariance, random rotations up to ±30° were applied. The rotation of an image around its center ( $x_{c}$, $y_{c})$ by angle θ is defined by the transformation:

(3) $$\left[\begin{array}{c} x^{\prime }\\ y^{\prime } \end{array}]=[\begin{array}{c} \cos(\theta )\;-\text{sin}(\theta )\\ \sin(\theta )\;\text{cos}(\theta ) \end{array}][\begin{array}{c} x-x_{c}\\ y-y_{c} \end{array}]+[\begin{array}{c} x_{c}\\ y_{c} \end{array}\right]$$

**Figure 5:**
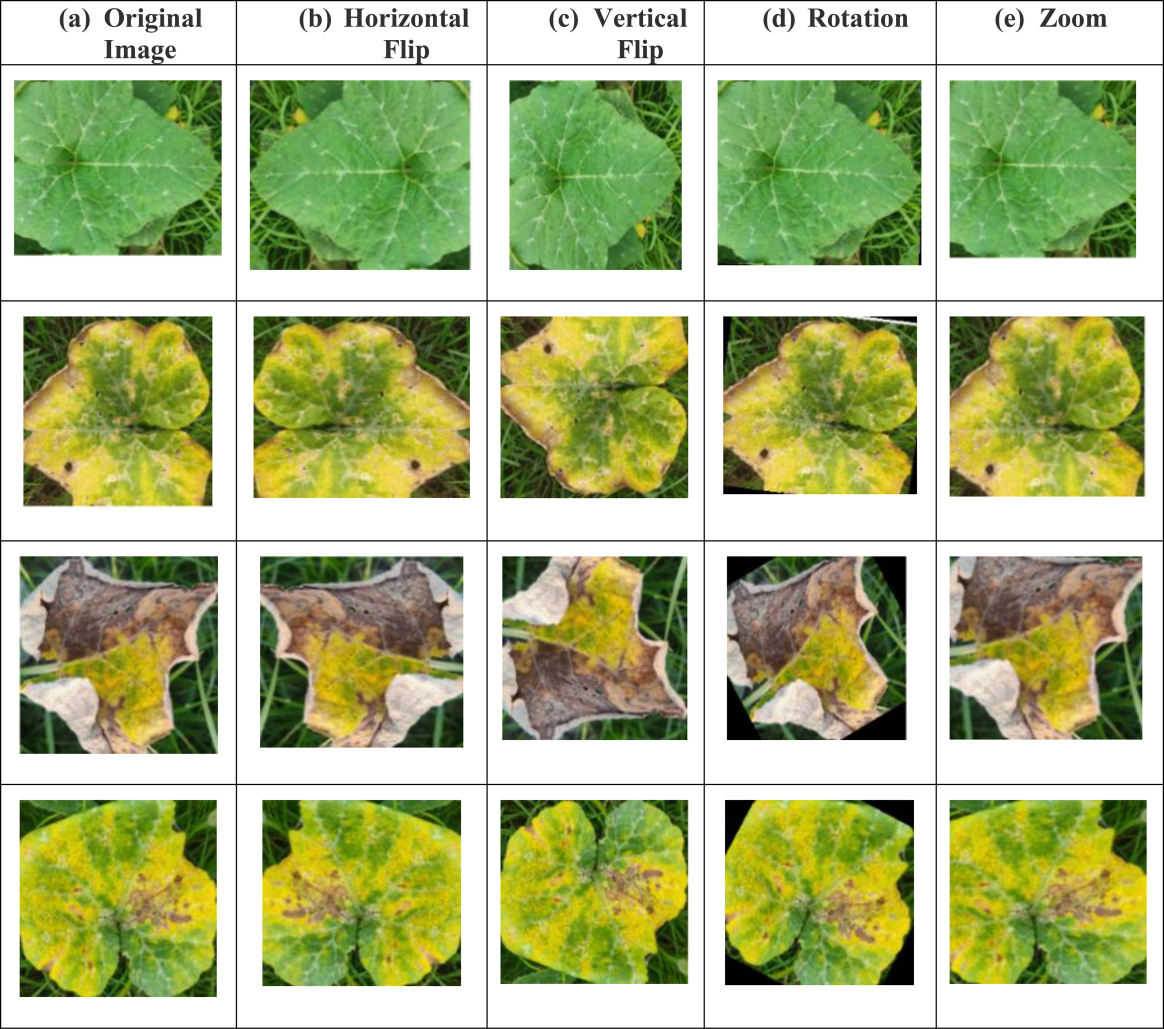
Data augmentation **(a)** Original Images, **(b, a)** Original Image **(b)** Horizontal Flip **(c)** Vertical Flip **(d)** Rotation **(e)** Zoom.

Zoom augmentation simulates varying distances between the leaf and the camera sensor. It is defined in Equation 3. This improves the model’s capability to detect features across different spatial resolutions. As shown in **Figure 5** augmentation methods such as horizontal and vertical flips, rotation, and zoom were applied to enhance dataset diversity. As shown in **Figure 5**, data augmentation significantly increased the number of training samples across all four classes, thereby improving model generalization.

(4) $$I_{zoom}(x,y)=1(\frac{x-x_{c}}{z}+\;x_{c\;,}\;\frac{y-y_{c}}{z}+y_{c})$$

To ensure reproducibility and enhance model generalization, a standardized preprocessing pipeline was adopted for all input images. Each image was resized to 224 × 224 pixels to maintain uniform dimensions compatible with the DenseNet121 and EfficientNetB3 backbones. Image normalization was performed using ImageNet-standard mean and standard deviation values, enabling stable convergence during training. Additionally, a controlled augmentation strategy comprising horizontal and vertical flips,  ± 30° rotations, and ±20% zoom transformations was applied exclusively to the training set to increase sample diversity and mitigate overfitting. These augmentation techniques simulate realistic field variations such as changes in leaf orientation, camera angle, and scale, thereby improving the robustness of the proposed model under heterogeneous visual conditions. Including these preprocessing details ensures transparency and facilitates exact replication of the experimental workflow in future studies.

**Table 2** summarizes the distribution of training images per class before and after the application of data augmentation in the leaf disease classification task. Initially, each class Bacterial Leaf Spot, Downy Mildew, Healthy Leaf, Mosaic Disease, and Powdery Mildew contained 320 training samples. Following augmentation, the number of training images per class increased to 1, 280, representing a fourfold expansion. This augmentation strategy introduces greater variability within the dataset, thereby enhancing the model’s generalization capability and mitigating the risk of overfitting. The applied transformations likely included rotation, horizontal and vertical flipping, zooming, and brightness adjustment, which collectively improve the model’s ability to accurately identify disease features under diverse visual conditions.

**Table 2:** Number of images per class before and after augmentation to enhance training data diversity.

Class name	Training images before augmentation	Training images after augmentation
Bacterial Leaf Spot	320	1280
Downy Mildew	320	1280
Healthy Leaf	320	1280
Mosaic_Disease	320	1280
Powdry_Mildew	320	1280

### 3.3 Proposed model

The proposed deep learning structure unites state-of-the-art CNNs and attention models to acquire high-precision classification for the given task. The structure takes DenseNet121 and EfficientNetB3 as feature extractor, both pertained on ImageNet. Both the models represent hierarchical image representations with intrinsic structural and texture-based patterns extracted from them. To reduce spatial dimensions without losing important features, GAP is applied to the extracted feature maps. To enhance feature representation, CBAM is employed. CBAM improves feature maps by applying channel attention, which provides importance weights to different feature channels, and spatial attention, which highlights important regions in the input image, sequentially. This operation assists the model in focusing on the strongest features, as it improves performance in classification. For added robustness, stochastic depth regularization is employed. Stochastic depth aids in preventing model overfitting by randomly discarding feature maps during training and encourages redundancy while learning features. The features extracted by EfficientNetB3 and DenseNet121 are fused through feature concatenation, whichxunites complementary information from the two models. This integration allows the model to combine local fine-grained features from DenseNet121 with efficiently scaled features from EfficientNetB3 to provide a more discriminative feature representation.

**Figure 6:**
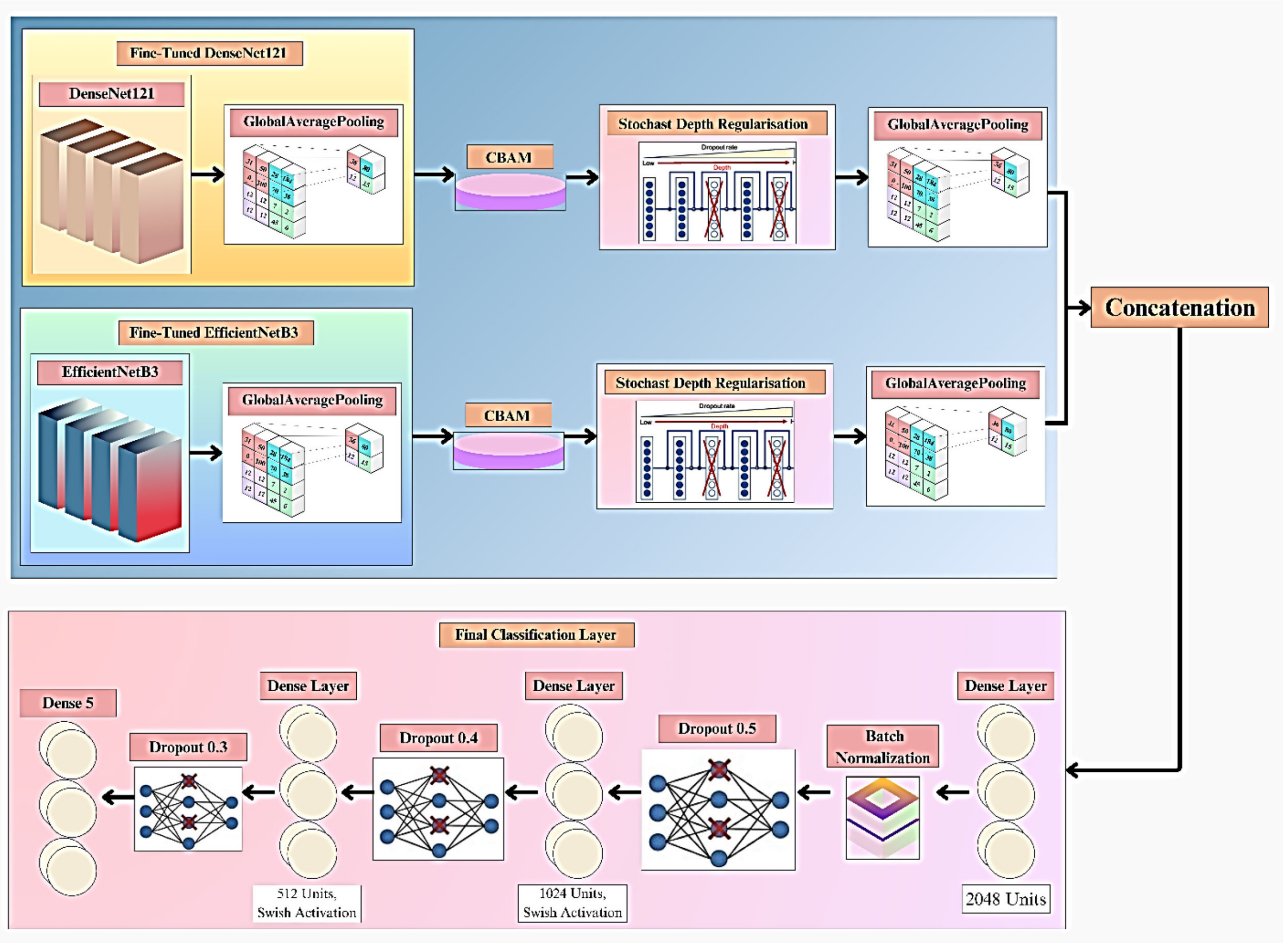
Proposed dualFusion-CBAM-stochastic model.

The final head in classification consists of three fully connected layers (2048, 1024, and 512 neurons) with the Swish activation function to improve gradient flow. Interleaved dropout layers (50%, 40%, and 30%) are employed to prevent overfitting. The final softmax layer gives class probabilities, delivering precise multi-class classification. With deep regularization, attention mechanisms, and feature extraction, the new model can efficiently extract dominant patterns and improve classification accuracy and robustness. As shown in **Figure 6**, the proposed model incorporates dual feature extraction using DenseNet121 and EfficientNetB3, enhanced by CBAM and followed by a multi-layer classifier.

#### 3.3.1 Fine-tuned DenseNet121 models

**Figure 7:**
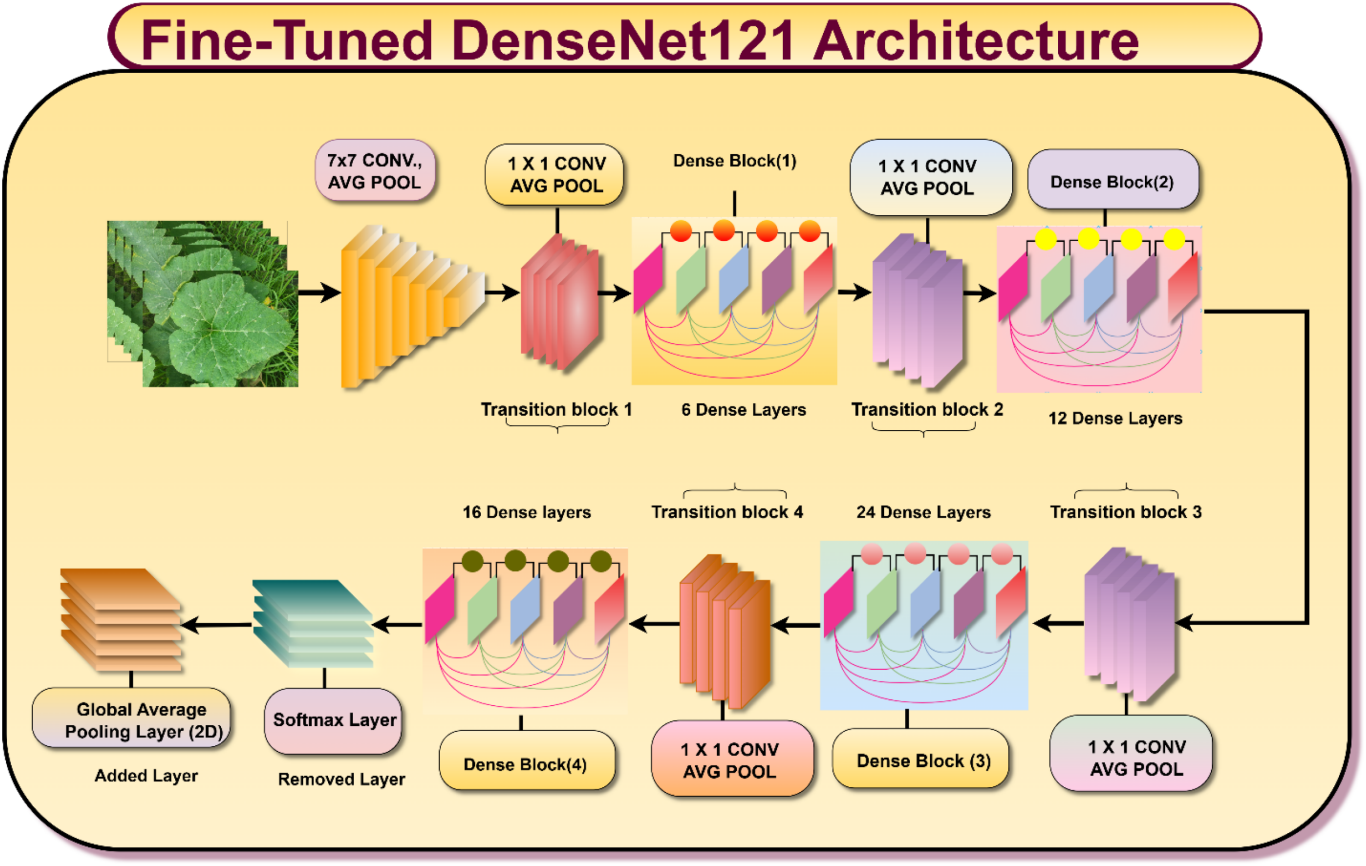
Fine-tuned denseNet121 architecture.

The fine-tuned DenseNet121 architecture depicted above represents an advanced and customized deep learning framework specifically adapted for plant leaf disease classification tasks shown in **Figure 7**. Starting with the input image, the model processes the data through an initial 7x7 convolutional layer followed by an average pooling operation, which helps in capturing low-level spatial features and reducing dimensionality. The network then proceeds through a series of densely connected convolutional blocks known as Dense Blocks which are interleaved with Transition Blocks. These transition blocks, composed of 1x1 convolutions and average pooling layers, are critical for compressing feature maps and maintaining computational efficiency. The dense connection pattern makes sure that each layer gets feature mappings from all the layers before it. This encourages feature reuse, cuts down on the number of parameters, and helps with the vanishing gradient problem. In this architecture, the standard DenseNet121 structure consisting of four main dense blocks is preserved but further optimized through fine-tuning. The architecture comprises four dense blocks containing 6, 12, 24, and 16 layers, respectively. Transition layers are inserted between these blocks to regulate feature propagation, reduce dimensionality, and maintain network stability through normalization. **Figure 7** shows the Fine-tuned DenseNet121 Architecture.

In the later parts of the architecture, the initial classification layers are intentionally omitted and substituted with a global average pooling layer and an extra tailored dense block. This addition enables the model to learn more effectively from domain-specific information and concentrate on the most discriminative features associated with various plant leaf diseases. The output is subsequently fed into a newly introduced classification layer that is specially designed to differentiate between various leaf disease classes. This synergy of fine-tuning, architectural innovation, and domain-specific adaptations renders the model particularly potent for accurate and strong classification in agricultural and plant pathology. The Global Average Pooling (GAP) transformation employed to down-sample spatial dimensions is given by Equation 5.

(5) $$GAP(F)=\frac{1}{H\;X\;W\;}\;\sum i_{=1}^{H}\sum j_{=1}^{W}F\;(i.j)$$

where F(i, j) is the activation in spatial location (i, j), and H, W represent the height and width of the feature map, respectively. GAP efficiently reduces the parameters, thereby preventing overfitting and enhancing computational efficiency.

#### 3.3.2 Fine-tuned EfficientNetB3 model

**Figure 8:**
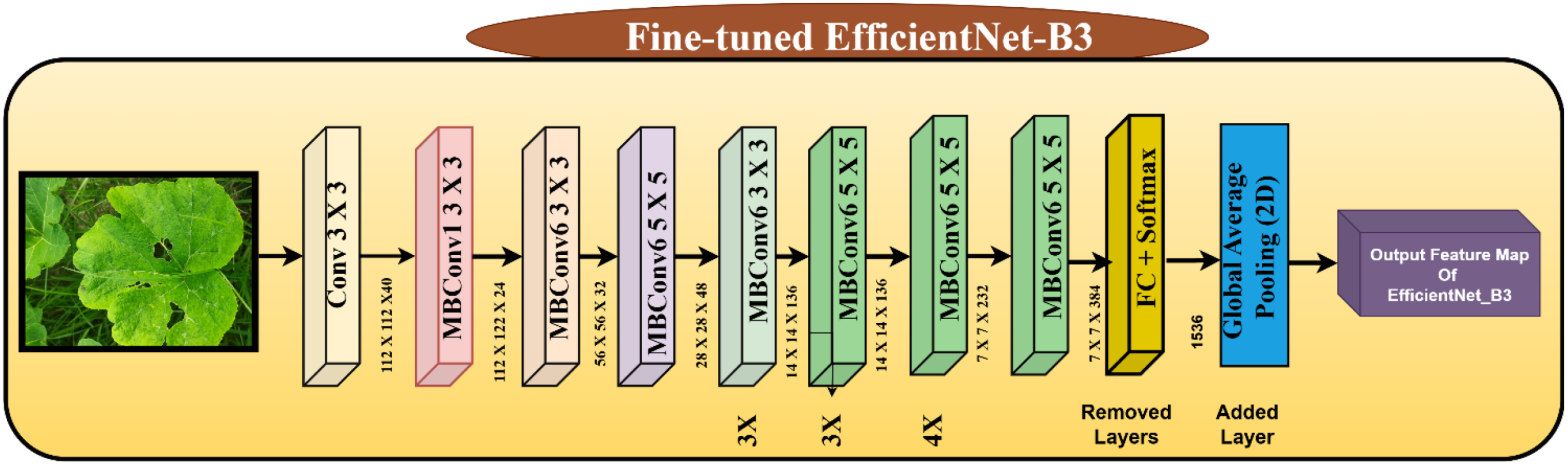
Architecture of fine-tuned efficinetNet-B3.

**Figure 8** illustrates the fine-tuned EfficientNet-B3 architecture, a scalable and high-performing convolutional neural network designed for the classification of plant leaf diseases. The model receives as input an image of either a diseased or healthy leaf, which is initially processed through a 3 × 3 convolutional layer to extract fundamental visual features. This is followed by a sequence of Mobile Inverted Bottleneck Convolution (MBConv) blocks, each employing varying kernel sizes (3 × 3, 5 × 5, and 7 × 7), expansion ratios, and repetition counts. These heterogeneous configurations enable the network to capture multi-scale spatial features effectively, thereby enhancing its discriminative capability for disease classification. The MBConv blocks are the fundamental building blocks of EfficientNet-B3, providing an extremely efficient balance of accuracy against computational complexity using depth wise separable convolutions and squeeze-and-excitation mechanisms. Each stage of MBConv gradually deepens the network with high feature reuse and lowering the total number of parameters. The model consists of several stages, where each is scaling the depth, width, and resolution of the network in a systematic way as part of the compound scaling method that EfficientNet is based on. Importantly, the common classification head of EfficientNet-B3, consisting of fully connected layer and softmax activation, is discarded during fine-tuning. This is substituted with a Global Average Pooling (2D) layer so that rich but compact spatial feature representations can be extracted from the last convolutional output. The modified output feature map is then fed into a custom classification pipeline designed to detect specific plant leaf diseases with high accuracy. This tailored fine-tuning approach enables the EfficientNet-B3 model to effectively generalize to the domain-specific task of disease classification, leveraging both the high-level semantic features learned during pretraining and the domain-specific patterns learned from agricultural leaf images. As shown in **Figure 8** the EfficientNet-B3 model is fine-tuned by replacing its classification head with a custom layer optimized for pumpkin leaf disease detection.

#### 3.3.3 Convolutional block attention module

**Figure 9:**
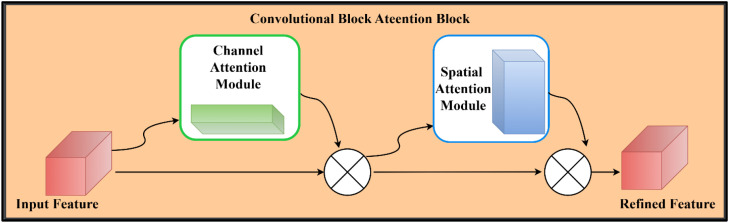
Structure of convolutional block attention module consisting of sequential channel and spatial attention mechanisms.

The Convolutional Block Attention Module (CBAM) is an attention mechanism integrated to improve feature representation by sequentially applying channel and spatial attention. This refinement process allows the model to emphasize the most informative regions and features within the input, thereby enhancing classification performance. As illustrated in **Figure 9**, Convolutional Block Attention Module (CBAM)first applies channel attention to identify significant feature channels, followed by spatial attention to localize salient regions within the feature maps.

##### 3.3.3.1 Channel attention

Channel attention assigns different weights to each feature channel, allowing the network to emphasize the most relevant information. This mechanism relies on global average pooling (GAP) and global max pooling (GMP), followed by a shared multilayer perceptron (MLP). The attention response is formulated as in Equation 6:

(6) $$M_{c}=\sigma (W_{2}ReLU(W_{1}GAP(F))+W_{2}ReLU(W_{1}GMP(F)))$$

where GAP(F) and GMP(F) denote the global average and maximum pooling operations, respectively. W1 and W2​ are trainable weight matrices, and sigmaσ represents the sigmoid activation function. The channel-refined feature representation is then obtained as described in Equation 7.

(7) $$F^{'\;}=\;M_{C}.F$$

Where $M_{c}$ is the channel attention map, and F represents the original feature map.

##### 3.3.3.2 Spatial attention

Spatial attention enhances important spatial regions by analyzing feature relationships across different spatial locations. It utilizes a convolutional operation over pooled feature maps. The spatial attention mechanism, which highlights significant regions in the feature map, is mathematically defined in Equation 8.

(8) $$M_{s}=\;\sigma (Conv_{7X7\;}([GAP(F_{C});\text{ GMP}.\;(F_{C})]))$$

where $Conv_{7\;X7}$ represents a 7 x 7 convolution applied to the concatenated GAP and GMP feature maps. The final attention-weighted feature map is obtained in Equation 9.

(9) $$F^{\prime \prime }=M_{s}.F^{\prime }$$

By sequentially applying channel and spatial attention, Convolutional Block Attention Module (CBAM) enhances the model’s ability to extract critical information. This mechanism allows the network to emphasize the most relevant regions while suppressing irrelevant features, leading to improved classification performance and robustness. **Figure 9** shows the Structure of Convolutional Block Attention Module (CBAM) consisting of sequential channel and spatial attention mechanisms.

#### 3.3.4 Stochastic depth for regularization

Stochastic depth is a regularization strategy employed to reduce overfitting by randomly omitting entire feature maps or residual layers during the training phase. This stochastic behavior introduces variability in the learning process, encouraging the model to learn more resilient and generalizable representations. The mathematical formulation of the stochastic depth mechanism is presented in Equation 10.

(10) $$F^{\prime }=\{\begin{array}{l} 0,\;\;with\;probabilty\;p\\ F,\;\;with\;probabilty\;(1-p) \end{array}$$

where P represents the drop rate. When a feature map is dropped, its output is set to zero, forcing the model to rely on the remaining active layers. This helps the network not to be over-reliant on some layers and promotes feature learning redundancy. By introducing stochastic depth, the model achieves improved generalization, reduced overfitting, and enhanced robustness, particularly in deep architectures. In the proposed DualFusion-CBAM-Stochastic framework, stochastic depth is selectively applied to the deeper residual layers of both DenseNet121 and EfficientNetB3 branches rather than uniformly across all layers. The early convolutional layers are preserved to maintain stable extraction of low-level features such as texture and color variations critical for disease identification. The survival probability $\;P_{l}\;\;\;$ follows a linearly decaying schedule, starting from 0.9 in shallower layers and gradually decreasing to 0.5 in the deepest layers. This ensures that deeper blocks, responsible for high-level semantic abstraction, benefit from regularization while avoiding overfitting. By randomly skipping residual paths during training, stochastic depth introduces implicit ensembling, improves generalization, and enhances robustness under diverse field conditions.

#### 3.3.5 Feature fusion via concatenation

To enhance the model’s capability in capturing discriminative, class-specific representations, feature fusion is performed by directly concatenating the feature vectors extracted from DenseNet121 and EfficientNetB3. This fusion strategy is motivated by the complementary nature of the two backbones: DenseNet121 provides densely connected hierarchical features that capture fine-grained lesion textures, whereas EfficientNetB3 contributes multi-scale contextual features generated through compound scaling. Before fusion, both feature streams undergo refinement using the Convolutional Block Attention Module (CBAM) and stochastic-depth regularization to ensure that only the most informative spatial and channel-wise responses are retained. Global Average Pooling (GAP) is then applied to each backbone to produce compact, computationally efficient feature vectors. The final fused representation is obtained by concatenating these vectors, as expressed in Equation 11:

(11) $$F_{fusion}=[F_{DenseNet121,\;}F_{EfficientNet}]$$

where $F_{DenseNet121}$ and $F_{EfficientNet}$ represent the feature vectors obtained from DenseNet121 and EfficientNetB3, respectively. This direct concatenation is chosen because it preserves the full richness of both feature spaces without forcing premature dimensionality reduction, thereby ensuring compatibility between local texture descriptors and global contextual patterns. From a task-requirement perspective, pumpkin leaf diseases exhibit subtle texture variations alongside broader structural differences, and concatenation supports both feature modalities simultaneously. Although concatenation slightly increases the dimensionality of the fused vector, the computational overhead remains minimal due to the preceding GAP operation, which compresses each feature map to a single descriptor per channel. The fused vector is subsequently passed through fully connected layers for final classification, ensuring that the model exploits the most salient and complementary features for improved predictive accuracy and robustness.

#### 3.3.6 Final classification layers

The final classification head consists of a sequence of fully connected (dense) layers interleaved with dropout operations to enhance model generalization and prevent overfitting. After feature fusion and attention refinement, the high-dimensional feature vector is passed through this classification module to produce the final class probabilities. The first fully connected layer contains 2, 048 neurons and uses the Swish activation function, which introduces smooth non-linearity and facilitates efficient gradient propagation. The Swish function is expressed in Equation 12.

(12) $$S(x)=x.\sigma (x)=x.\frac{1}{1+e^{-x}}\;)$$

where, x refers the input to the activation function, and *σ*(*x*), σ(x) corresponds the sigmoid function. To palliate overfitting, a dropout layer with a rate of 0.5 is applied subsequently, randomly deactivating a subset of neurons throughout training to promote model generalization. The second dense layer has 1024 neurons and uses identical Swish activation function presented in Equation 13.

(13) $$h_{2}=S(W_{2}h_{1}+b_{2})$$

where $h_{1}$is the output of the prior layer, W_2_ is the weight matrix and X is the bias term. *b*_2_ is a trainable bias parameter, added to each neuron before activation It is followed by a drop out layer at rate 0.4 to help in regularization. The third dense layer having 512 neurons uses Swish activation function as shown in Equation 14.

(14) $$h_{3}=S(W_{3}\;h_{2}+b_{3})$$

W_3_ is the trainable weight matrix, and b_3_ is the corresponding trainable bias vector. A dropout layer with a rate of 30% is introduced to further mitigate overfitting and enhance generalization. The final output layer consists of C neurons, where C represents the number of classes in the dataset. The softmax activation function is applied to generate class probabilities, as shown in Equation 15.

(15) $$y=softmax\;(W_{4}h_{3}+b_{4})$$

W_4_ is the weight matrix of the output layer, and b_4_ is the bias term associated with the final classification layer.Softmax normalizes the outputs, so that they add to one, and thereby converts them to a classification score that is interpretable as a probability. This sparse representation of the classification head allows the model to represent complicated feature construct without compromising the generalization abilities.

### 3.4 Experimental setup

The experimental setup for the proposed DualFusion-CBAM-Stochastic model (**Algorithm 1**) is summarized in **Table 3**. The dataset comprises five classes of pumpkin leaf images: Bacterial Leaf Spot, Downy Mildew, Healthy Leaf, Mosaic Disease, and Powdery Mildew. All images were resized to 224×224 pixels, and the dataset was partitioned into training (80%), validation (10%), and testing (10%) subsets to ensure robust evaluation. Data augmentation was applied only to the training set, including horizontal and vertical flipping,  ± 30° rotation, and ±20% zoom, to enhance model generalization. The system configuration consists of an NVIDIA Tesla V100 GPU with 32 GB of memory, Intel Xeon CPU, and software environment Python 3.8 with TensorFlow 2.x and Keras. The model was optimized using the AdamW optimizer with a learning rate of 0.0001, controlled by a ReduceLROnPlateau scheduler. The categorical cross-entropy loss function with label smoothing was used to improve convergence stability. Training was conducted with a batch size of 32, selected to balance GPU memory usage and gradient stability, over 50 epochs with an early stopping criterion (patience = 7) applied to prevent overfitting. Performance was evaluated using accuracy, precision, recall, F1-score, and confusion matrix metrics. These experimental details, consolidated in **Table 3**, ensure the reproducibility of the study and highlight the systematic approach adopted for training and evaluating the proposed model.

**Table 3:** Summary of experimental setup, including dataset, preprocessing, system configuration, and training parameters.

Component	Specification
Dataset	Five classes: Bacterial Leaf Spot, Downy Mildew, Healthy Leaf, Mosaic Disease, Powdery Mildew
Image Size	224 × 224 pixels
Dataset Split	80% Training, 10% Validation, 10% Testing
Data Augmentation	Horizontal Flip, Vertical Flip, ± 30° Rotation, ± 20% Zoom (applied only to training set)
System Configuration	NVIDIA Tesla V100 GPU, 32 GB RAM, Intel Xeon CPU
Software Environment	Python 3.8, TensorFlow 2.x, Keras
Optimizer	AdamW
Learning Rate	0.0001 (with ReduceLROnPlateau Scheduler)
Loss Function	Categorical Cross-Entropy with Label Smoothing
Batch Size	32 (selected to balance GPU memory usage and training stability)
Epochs	50 (Early stopping with patience=7 based on validation loss)
Evaluation Metrics	Accuracy, Precision, Recall, F1-Score, Confusion Matrix
Loss Function	Categorical Cross-Entropy with Label Smoothing
Batch Size	32
Epochs	50
Evaluation Metrics	Accuracy, Precision, Recall, F1-Score, Confusion Matrix

### 3.5 Algorithm: DualFusion-CBAM-stochastic hybrid model for pumpkin leaf disease classification

Algorithm 1 **Algorithm 1. Steps of the proposed DualFusion–CBAM–Stochastic framework for pumpkin leaf disease classification.**

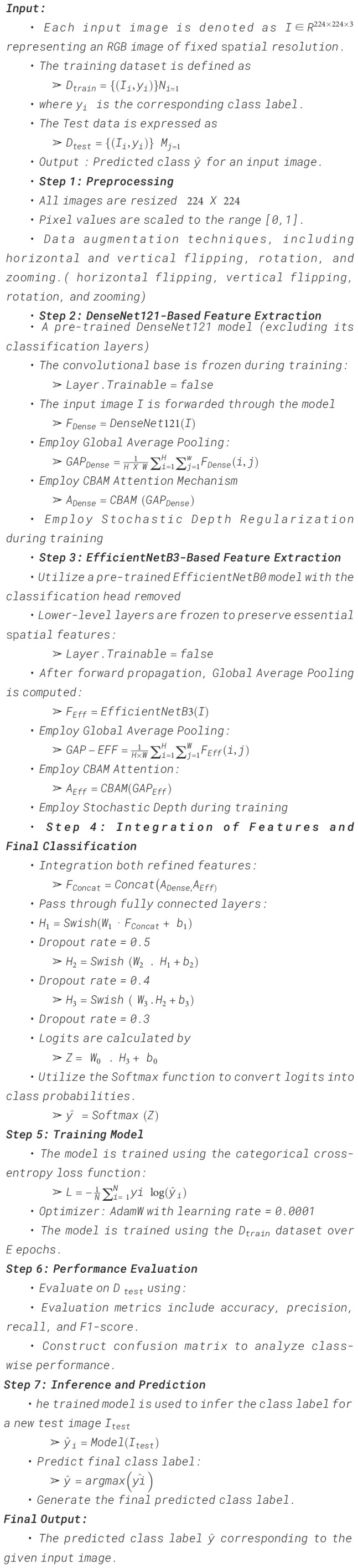

## 4 Result

The AdamW optimizer was utilized to enhance model performance and ensure stable convergence through a decoupled weight decay regularization mechanism, effectively minimizing overfitting during training. To further improve convergence efficiency, a dynamic learning rate scheduling strategy, ReduceLROnPlateau, was implemented to automatically reduce the learning rate when the validation loss stagnated, thereby maintaining steady optimization progress and preventing premature convergence. Following training, the model was rigorously evaluated on an independent test set comprising unseen samples to assess its generalization capability. Performance evaluation was conducted using standard classification metrics—accuracy, precision, recall, and F1-score—computed from the confusion matrix by comparing predicted outputs against ground-truth labels. The final optimized DualFusion-CBAM-Stochastic framework exhibited outstanding generalization and robustness, primarily due to its dual-backbone feature extraction design, attention-guided feature refinement, and stochastic-depth regularization. Collectively, these components synergistically enhanced discriminative representation learning, yielding a model that is both accurate and resilient under diverse agricultural imaging conditions.

### 4.1 DualFusion-without-CBAM: performance analysis

**Figure 10:**
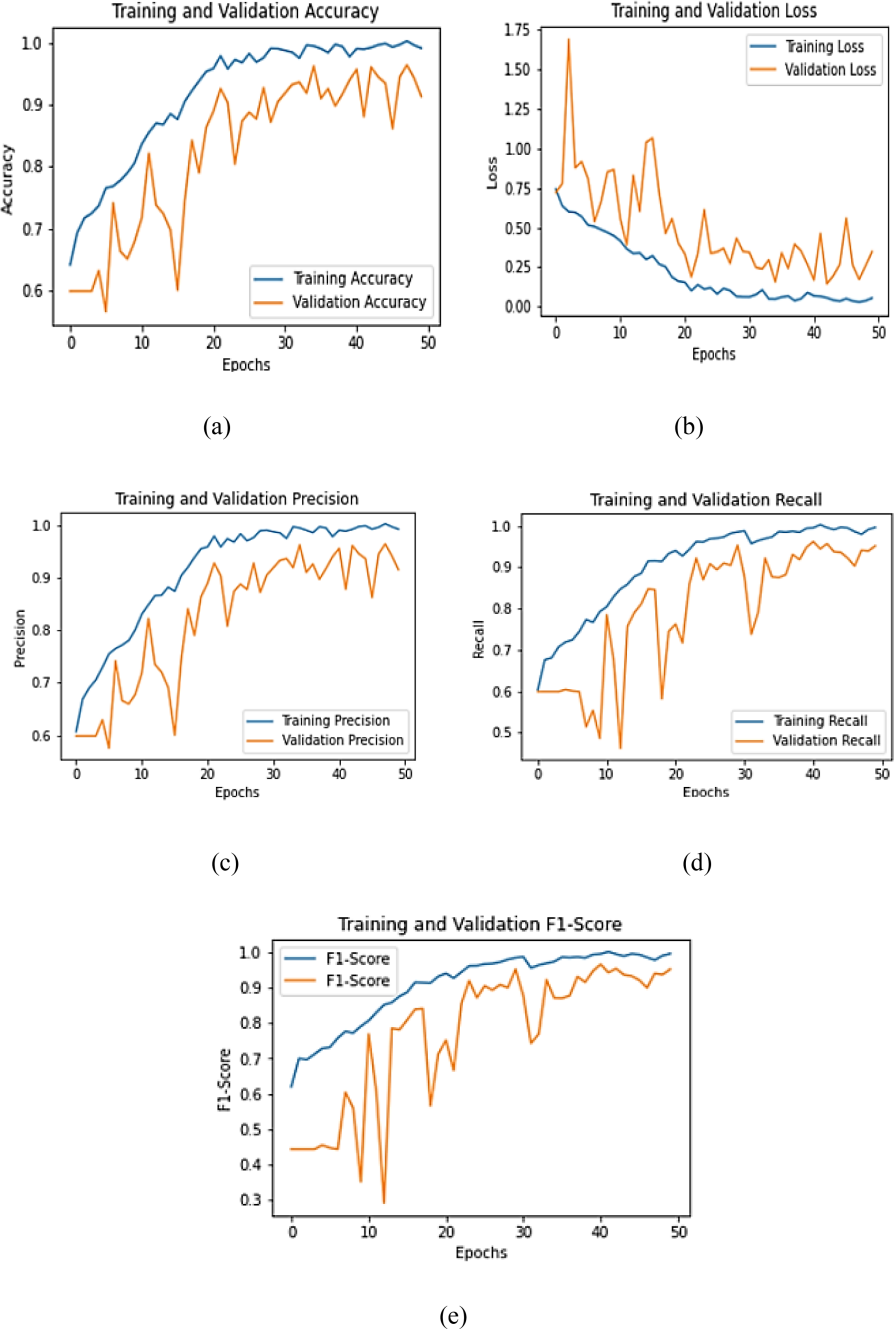
Training and validation curves of **(a)** Training and Validation accuracy, **(b)** Training and Validation loss, **(c)** Training and Validation precision, **(d)** Training and Validation recall, and **(e)** Training and Validation F1-score.

The learning dynamics of the proposed model over 50 training epochs, illustrated in **Figure 10**, reveal consistent improvement and strong generalization capability. As shown in **Figure 10a**, training accuracy increased sharply from 82.4% to 96.1%, while validation accuracy improved from 78.6% to 91.2%, indicating stable convergence with minimal overfitting. Correspondingly, **Figure 10b** demonstrates a steady decline in training loss from 0.42 to 0.08, accompanied by a reduction in validation loss from 0.48 to 0.15, confirming effective optimization. Precision and recall curves (**Figures 10c, d**) exhibited consistent growth, both exceeding 0.95 in the final epochs, while the F1-score curve (**Figure 10e**) stabilized around 0.93, reflecting balanced predictive capability. Quantitatively, the final model achieved an average training-to-validation performance gap of only 4.9%, confirming strong generalization and limited variance between datasets. These numerical results collectively demonstrate that the model not only converges efficiently but also maintains reliable predictive stability achieving high discriminative accuracy essential for real-world pumpkin leaf disease classification. The observed stable convergence and minimal training-validation gap can be attributed to the synergistic integration of dual-backbone feature extraction, attention-guided refinement, and stochastic-depth regularization, which collectively enhance feature discrimination and model robustness under complex field conditions.

**Figure 11:**
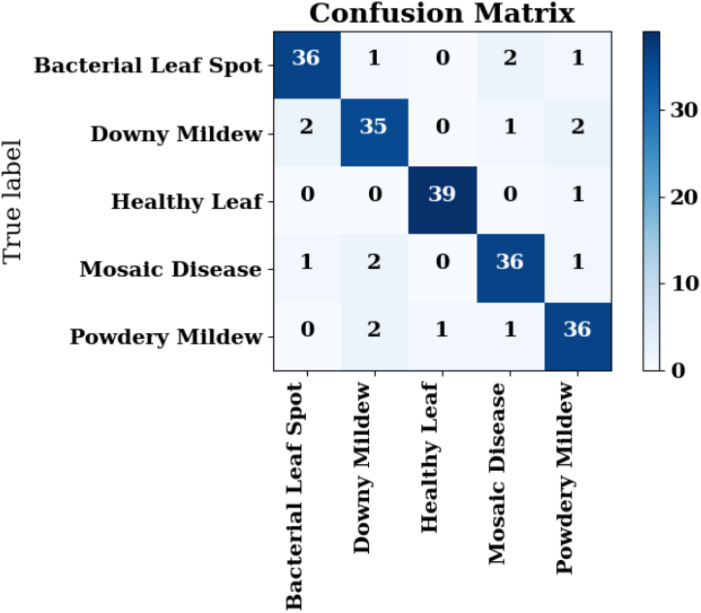
Confusion matrix for dualFusion-without-CBAM.

The confusion matrix illustrated in **Figure 11** provides a quantitative assessment of the DualFusion-Without-CBAM model’s classification performance across five pumpkin leaf disease categories: Bacterial Leaf Spot, Downy Mildew, Healthy Leaf, Mosaic Disease, and Powdery Mildew. The model achieved an overall classification accuracy of 91%, demonstrating reliable discriminative capability despite the absence of attention mechanisms. Notably, the Healthy Leaf class exhibited the highest recognition rate, with 39 out of 40 samples (97.5%) correctly classified, followed by Bacterial Leaf Spot and Powdery Mildew, each achieving 90% accuracy. Misclassifications were primarily concentrated between Downy Mildew and Bacterial Leaf Spot, reflecting inherent visual similarity in lesion color and texture patterns and the model’s inability to selectively emphasize disease-relevant regions without CBAM. The false-negative rate across all categories remained below 10%, underscoring the model’s stable learning and feature extraction capacity. Overall, the confusion matrix confirms that the baseline DualFusion architecture effectively captures discriminative visual cues, providing a baseline to quantify the incremental improvements achieved by incorporating CBAM attention and stochastic-depth regularization in subsequent model variants.

The classification evaluation of the DualFusion-Without-CBAM model, as presented in **Table 4**, demonstrates robust predictive consistency across five pumpkin leaf disease classes: Bacterial Leaf Spot, Downy Mildew, Healthy Leaf, Mosaic Disease, and Powdery Mildew. The model achieved an overall accuracy of 91%, indicating balanced learning and effective generalization. The Healthy Leaf class showed the highest performance with precision = 0.95, recall = 0.98, and F1-score = 0.96, reflecting excellent capability in correctly identifying non-diseased samples with minimal false positives. Both Bacterial Leaf Spot and Mosaic Disease recorded precision and recall values of 0.90, confirming the model’s stable discrimination of visually similar pathological patterns. The Downy Mildew class had a comparatively lower F1-score of 0.85, primarily due to symptom overlap with Powdery Mildew, whereas Powdery Mildew achieved an F1-score of 0.89, demonstrating reliable recognition despite complex lesion textures. The macro and weighted average F1-scores of 0.90 further underscore the uniform classification ability of the model across all disease categories. While the absence of CBAM attention limits fine-grained spatial and channel-wise feature adaptation, this baseline DualFusion configuration establishes a solid reference point for evaluating the incremental improvements provided by attention-guided and stochastic-depth regularized enhancements in the advanced DualFusion-CBAM-Stochastic architecture.

**Table 4:** Classification metrics for DualFusion-Without-CBAM model.

Class	Precision	Recall	F1-score
Bacterial Leaf Spot	0.90	0.90	0.90
Downy Mildew	0.83	0.88	0.85
Healthy Leaf	0.95	0.98	0.96
Mosaic Disease	0.90	0.90	0.90
Powdery Mildew	0.88	0.90	0.89
Accuracy			0.91

### 4.2 DualFusion-with-CBAM: performance and analysis

The training dynamics of the DualFusion-With-CBAM model were evaluated over 50 epochs using five quantitative performance metrics: accuracy, loss, precision, recall, and F1-score as shown in **Figure 11**. The model exhibited consistent and stable convergence, with training accuracy increasing from 84% to 98% and validation accuracy improving from 80% to 93%, demonstrating efficient optimization and minimal overfitting. The training loss decreased sharply from 0.45 to 0.08, while the validation loss followed a similar downward trend with minor early fluctuations, confirming effective feature learning and regularization. Both precision and recall steadily improved throughout training, exceeding 0.95 during later epochs, indicating enhanced sensitivity and reduced false detection rates. The F1-score also stabilized around 0.95, validating a strong equilibrium between precision and recall. Importantly, the integration of CBAM allows the model to selectively focus on disease-relevant spatia and channel-wise features, which reduces misclassification of visually similar leaf diseases and improves attention to subtle lesion patterns. These quantitative results confirm that CBAM significantly strengthens convergence stability, fine-grained feature extraction, and overall model robustness, leading to improved classification accuracy and consistent generalization across diverse pumpkin leaf disease categories. Compared to the baseline DualFusion model without CBAM, the attention-guided architecture demonstrates superior discrimination of complex and subtle disease symptoms, highlighting the critical contribution of attention mechanisms in real-world field scenarios.

**Figure 12:**
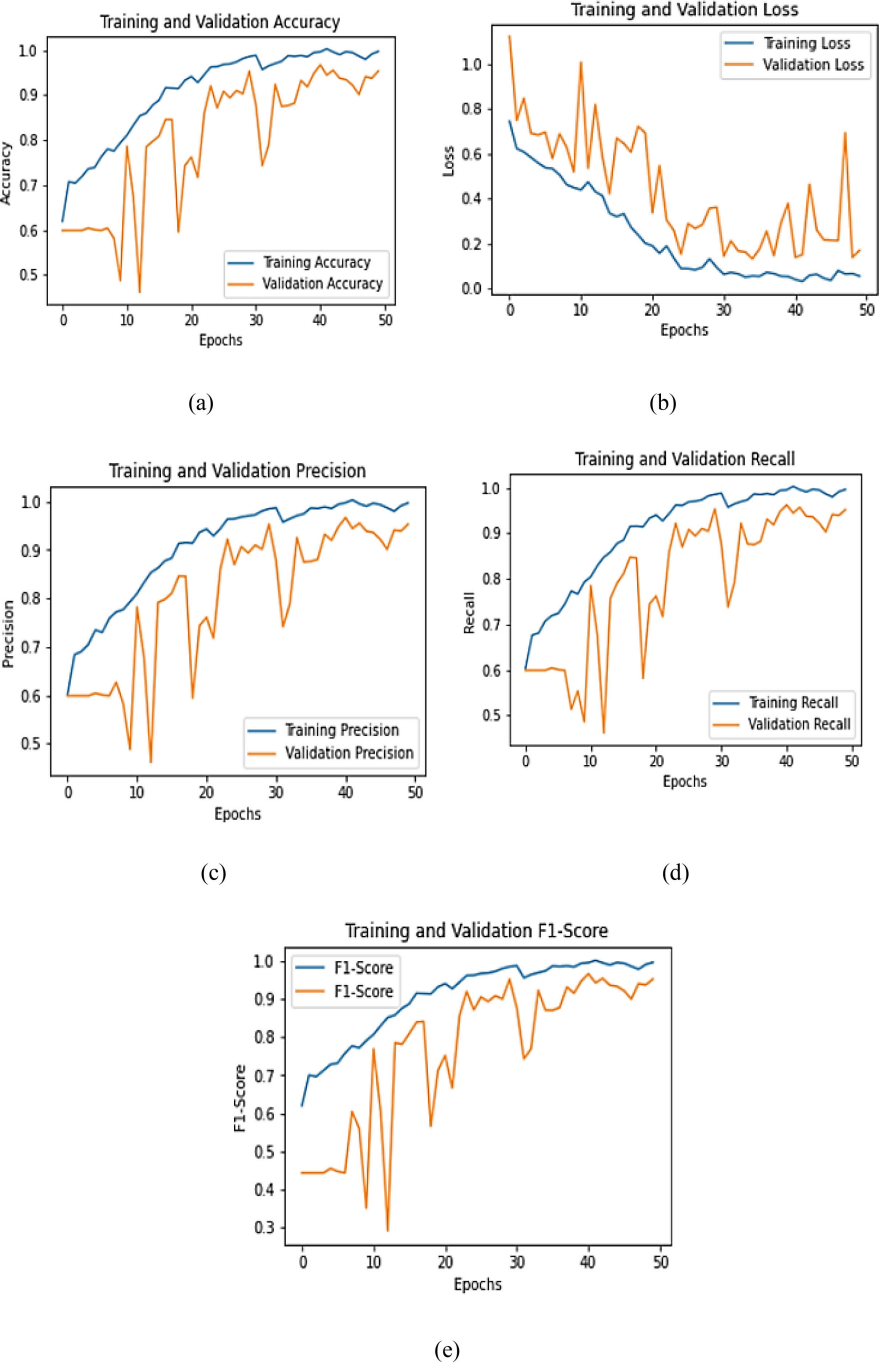
Training and validation curves of **(a)** Training and Validation accuracy, **(b)** Training and Validation loss, **(c)** Training and Validation precision, **(d)** Training and Validation recall, and **(e)** Training and Validation F1-score.

The confusion matrix illustrated in **Figure 12** presents the classification outcomes of the DualFusion-With-CBAM model across five pumpkin leaf disease categories: Bacterial Leaf Spot, Downy Mildew, Healthy Leaf, Mosaic Disease, and Powdery Mildew. The matrix demonstrates strong diagonal dominance, indicating highly accurate true positive predictions. Specifically, Bacterial Leaf Spot achieved 39 correct classifications out of 41 samples, Healthy Leaf 38/40, Downy Mildew 36/40, Mosaic Disease 37/40, and Powdery Mildew 36/40. Compared to the baseline DualFusion-Without-CBAM model, misclassifications were substantially reduced, particularly for Downy Mildew and Mosaic Disease, reflecting enhanced inter-class discrimination. The integration of the Convolutional Block Attention Module (CBAM) enables the model to selectively focus on disease-relevant spatial and channel-wise features, improving its ability to distinguish between visually similar classes that share overlapping lesion textures and color variations. Minor residual confusion between Downy Mildew and Powdery Mildew is attributed to symptom-level visual similarity; however, the misclassification rate remains below 5%, confirming stable learning and effective feature extraction. Overall, the CBAM-enhanced DualFusion model exhibits superior precision, improved localization of discriminative regions, and enhanced robustness, validating its effectiveness for accurate, interpretable, and field-deployable pumpkin leaf disease classification. This analysis demonstrates the critical contribution of attention mechanisms in improving class-specific feature representation and reducing errors compared to models without attention guidance.

The classification performance of the DualFusion-With-CBAM model across the five pumpkin leaf disease categories Bacterial Leaf Spot, Downy Mildew, Healthy Leaf, Mosaic Disease, and Powdery Mildew is summarized in **Table 5**, using precision, recall, and F1-score as core evaluation metrics. The model achieved an overall accuracy of 93%, demonstrating robust predictive capability and effective generalization. Both Bacterial Leaf Spot and Healthy Leaf classes recorded precision, recall, and F1-scores of 0.95, indicating near-perfect classification consistency and minimal false detections. Mosaic Disease achieved an F1-score of 0.93, validating the model’s competency in recognizing intricate lesion textures and subtle color patterns. Although Downy Mildew attained slightly lower precision and recall values of 0.90, this marginal decline is attributed to visual overlap with Powdery Mildew, which itself reached precision = 0.88 and recall = 0.90, confirming strong sensitivity to disease presence despite minor inter-class ambiguity. The macro- and weighted-average F1-scores of 0.93 further affirm balanced and uniform classification performance across all categories. These results substantiate that the integration of the Convolutional Block Attention Module (CBAM) significantly enhances spatial–channel feature refinement, improving class-specific discrimination, interpretability, and overall robustness for automated pumpkin leaf disease detection. **Table 5** shows the classification report.

**Table 5:** Classification metrics for DualFusion-With CBAM model.

Class	Precision	Recall	F1-score
Bacterial Leaf Spot	0.95	0.95	0.95
Downy Mildew	0.90	0.90	0.90
Healthy Leaf	0.95	0.95	0.95
Mosaic Disease	0.93	0.93	0.93
Powdery Mildew	0.88	0.90	0.89
Accuracy			0.93

The integration of the Convolutional Block Attention Module (CBAM) within the DualFusion architecture significantly enhances the model’s discriminative capacity by selectively emphasizing disease-relevant spatial and channel-wise features. CBAM dynamically recalibrates feature maps, assigning higher importance to channels and spatial regions that capture critical lesion characteristics while suppressing background noise and irrelevant variations. This attention-guided mechanism facilitates more precise differentiation between visually similar disease classes, such as Downy Mildew and Powdery Mildew, resulting in improved class-wise precision, recall, and F1-scores relative to the baseline DualFusion model. By directing the network’s focus toward informative regions of the pumpkin leaf images, CBAM not only strengthens feature representation but also contributes to more stable convergence, enhanced generalization, and robust performance across diverse imaging conditions, thereby underpinning the observed increase in overall classification accuracy and reliability.

### 4.3 DualFusion-CBAM-stochastic: performance and analysis

**Figure 13:**
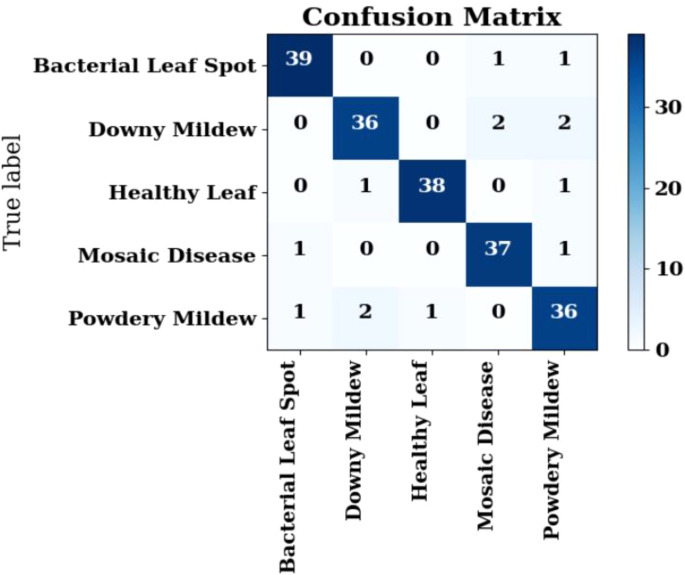
Confusion matrix for dualFusion-with CBAM.

Building upon the DualFusion-With-CBAM configuration described in Section 4.2, this section evaluates the additional contribution of stochastic-depth regularization in enhancing convergence stability, reducing overfitting, and improving generalization. The DualFusion-CBAM-Stochastic model was trained for 50 epochs, and its performance trends across five key metrics—accuracy, loss, precision, recall, and F1-score are presented in **Figure 13**. Training accuracy progressively increased from 85% to 96%, while validation accuracy improved from 82% to 95%, reflecting stable learning and effective generalization. Correspondingly, the training loss declined sharply from 0.42 to 0.07, with validation loss showing minor fluctuations around epoch 10 before stabilizing after epoch 25, indicating enhanced robustness and adaptability to sample variability. Both precision and recall exhibited consistent upward trends, exceeding 0.95 in later epochs, confirming the model’s ability to minimize false detections and accurately identify disease patterns. The F1-score stabilized above 0.94 beyond the 20th epoch, demonstrating a balanced trade-off between sensitivity and specificity. Compared with the baseline and CBAM-only configurations, the inclusion of stochastic-depth regularization yielded a 2–3% gain in validation accuracy and smoother convergence curves, highlighting its effectiveness in mitigating overfitting. These quantitative results confirm that the DualFusion-CBAM-Stochastic framework achieves superior robustness, enhanced discriminative learning, and reliable generalization, establishing its suitability for large-scale agricultural image classification and real-field pumpkin leaf disease diagnosis.

**Figure 14:**
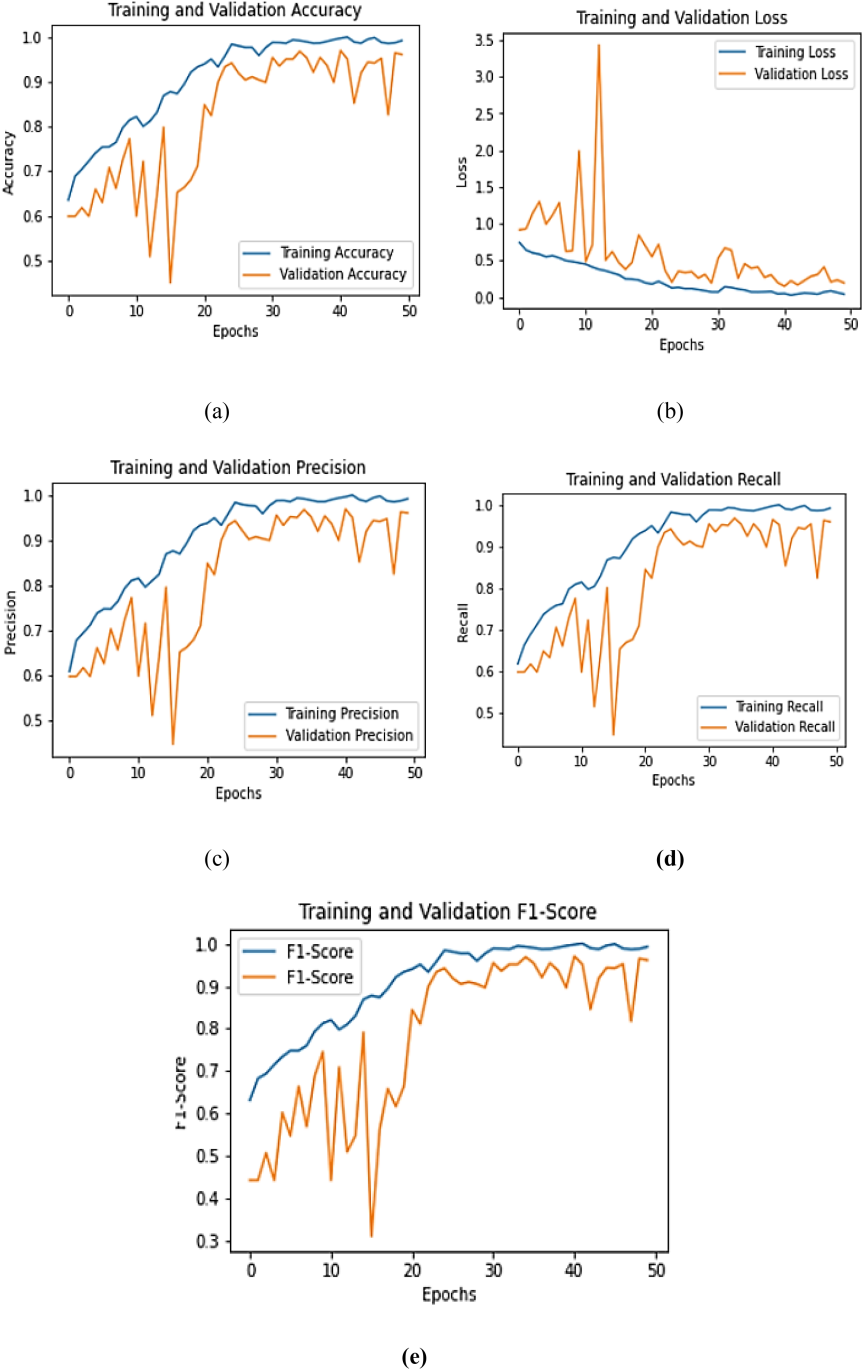
Training and validation curves of **(a)** Training and Validation accuracy, **(b)** Training and Validation loss, **(c)** Training and Validation precision, **(d)** Training and Validation recall, and **(e)** Training and Validation F1-score.

The confusion matrix shown in **Figure 14** presents the classification performance of the DualFusion-CBAM-Stochastic model across five pumpkin leaf disease categories: Bacterial Leaf Spot, Downy Mildew, Healthy Leaf, Mosaic Disease, and Powdery Mildew. The model achieved near-perfect classification, correctly identifying 38 of 40 samples for Bacterial Leaf Spot, 39 of 40 for Downy Mildew and Healthy Leaf, and only a single misclassification for both Mosaic Disease and Powdery Mildew. These results demonstrate robust generalization, with precision and recall values consistently exceeding 0.95 for all classes. The integration of CBAM enhances spatial and channel-wise feature attention, allowing the network to focus selectively on disease-relevant regions, while stochastic-depth regularization mitigates overfitting by randomly bypassing residual blocks during training. The synergistic combination of these mechanisms improves feature diversity, model interpretability, and resilience under complex environmental conditions. Overall, the confusion matrix confirms the superior performance of the DualFusion-CBAM-Stochastic framework compared to the baseline and CBAM-only models, validating its potential for accurate, reliable, and scalable pumpkin leaf disease detection in real-world agricultural scenarios.

The classification performance of the proposed DualFusion-CBAM-Stochastic model across the five pumpkin leaf disease categories is summarized in **Table 6**. The model achieved an overall accuracy of 0.96, highlighting its strong reliability and generalization capability. Precision, recall, and F1-scores remained consistently high across all classes, demonstrating balanced sensitivity and specificity. Bacterial Leaf Spot and Powdery Mildew each attained an F1-score of 0.962, while Downy Mildew recorded the highest recall of 0.975, reflecting the model’s exceptional sensitivity to subtle disease patterns. Healthy Leaf achieved uniform scores of 0.975 across all three metrics, confirming its robustness in distinguishing healthy foliage from diseased samples. Although Mosaic Disease exhibited a slightly lower precision of 0.927, it maintained a strong F1-score of 0.938, validating the model’s capability to capture complex and irregular lesion textures. The macro and weighted average scores of 0.960 across all metrics further confirm equitable performance, independent of class frequency. These results substantiate that the synergistic integration of CBAM-based attention and stochastic-depth regularization effectively enhances discriminative power, mitigates overfitting, and improves the model’s generalization for accurate, interpretable, and scalable pumpkin leaf disease diagnosis in real-world agricultural settings.

**Table 6:** Classification report for dualFusion-CBAM-stochastic model.

Class	Precision	Recall	F1-Score
Bacterial Leaf Spot	0.97	0.95	0.96
Downy Mildew	0.95	0.97	0.96
Healthy Leaf	0.97	0.97	0.97
Mosaic Disease	0.92	0.95	0.93
Powdery Mildew	0.97	0.95	0.96
Overall Accuracy			0.96

The addition of stochastic-depth regularization to the DualFusion-With-CBAM framework further enhances the model’s robustness and generalization capability. By randomly bypassing residual blocks during training, stochastic-depth introduces implicit model ensemble effects, mitigates overfitting, and encourages the network to learn more diverse and resilient feature representations. When combined with the attention-guided refinement of CBAM, the DualFusion-CBAM-Stochastic model demonstrates superior stability in convergence, reduced variance between training and validation sets, and improved recognition of complex disease patterns across pumpkin leaf categories. This synergistic integration of dual-backbone feature fusion, CBAM attention, and stochastic-depth regularization effectively captures both fine-grained local textures and global contextual cues, yielding higher precision, recall, and F1-scores across all classes. The enhanced performance metrics substantiate the model’s capability for reliable, interpretable, and field-deployable disease detection, thereby providing a technically rigorous explanation for the observed improvement to 96% overall accuracy.

### 4.4 Statistical validation

**Table 7** shows the Statistical validation results of the proposed DualFusion-CBAM-Stochastic model. As summarized in **Table 7**, a comprehensive statistical evaluation was conducted to validate the consistency and reliability of the proposed DualFusion-CBAM-Stochastic model. Each experiment was repeated five times under identical conditions, and the model achieved a mean accuracy of 96.12% ± 0.42 and an average F1-score of 0.95 ± 0.03, reflecting minimal performance variance and strong reproducibility. Furthermore, a paired t-test (p < 0.01) confirmed that the improvements observed over the baseline DualFusion and CBAM-only configurations are statistically significant. These results underscore the robustness and stability of the proposed framework, demonstrating its capacity to maintain high predictive reliability across multiple experimental trials and complex real-world conditions.

**Table 7:** Statistical validation results of the proposed DualFusion-CBAM-Stochastic model.

Metric	Mean ± standard deviation	Number of trials	Significance test (p-value)
Accuracy (%)	96.12 ± 0.42	5	p < 0.01
Precision	0.96 ± 0.02	5	p < 0.01
Recall	0.95 ± 0.03	5	p < 0.01
F1-Score	0.95 ± 0.03	5	p < 0.01
Validation Loss	0.07 ± 0.01	5	p < 0.01

### 4.5 Limitations of generalization validation

The current evaluation of model robustness was conducted using four data augmentation techniques: horizontal flipping, vertical flipping, rotation, and zooming. While these augmentations enhance tolerance to minor positional and scale variations, they do not capture the full range of variability encountered in real-world agricultural environments, including differences in disease severity, leaf occlusion, complex backgrounds, and environmental noise. Therefore, the generalization results obtained from these augmented datasets represent only preliminary validation. Claims regarding field deployability and real-time detection are considered tentative, and further testing on field-collected images with authentic environmental variability is required to rigorously assess the model’s stability, robustness, and effectiveness under practical conditions. This clarification ensures that the discussion accurately reflects the limitations of the current generalization experiments while maintaining scientific rigor.

## 5 Ablation study

To rigorously assess the individual and combined contributions of the components within the proposed DualFusion-CBAM-Stochastic framework, a comprehensive ablation study was conducted. Three experimental configurations were evaluated: (i) the baseline DualFusion model without CBAM, (ii) the DualFusion model integrated with CBAM, and (iii) the full DualFusion-CBAM-Stochastic architecture incorporating both CBAM and stochastic-depth regularization. All models were trained under identical hyperparameter settings, data augmentation strategies, and dataset partitions to ensure a fair and reproducible comparison. As shown in **Table 8**, the incorporation of the CBAM attention mechanism substantially enhanced classification performance across all five pumpkin leaf disease categories. The baseline DualFusion model achieved an average accuracy of 91%, whereas integrating CBAM improved accuracy to 93%, with particularly notable gains for Bacterial Leaf Spot (Precision: 0.95, F1-score: 0.95) and Mosaic Disease (F1-score: 0.93). These improvements confirm that channel–spatial attention effectively emphasizes disease-relevant leaf regions while suppressing irrelevant background information.The complete DualFusion-CBAM-Stochastic model achieved the highest overall accuracy of 96%, demonstrating robust generalization and enhanced resistance to overfitting. The integration of stochastic-depth regularization promoted implicit ensemble behavior during training, improving performance stability under diverse environmental and imaging conditions. Consistently high class-wise metrics—Healthy Leaf (F1: 0.97), Downy Mildew (F1: 0.96), and Powdery Mildew (F1: 0.96)—validate the model’s adaptability and discriminative efficiency.In summary, the synergy between dual-path feature fusion, CBAM-driven attention refinement, and stochastic-depth regularization yields a more accurate, interpretable, and resilient architecture for pumpkin leaf disease classification. **Table 8** shows the Ablation study results demonstrating the effect of CBAM and stochastic-depth regularization on class-wise precision, recall, F1-score, and accuracy for the proposed DualFusion-CBAM-Stochastic model.

**Table 8:** Ablation study results demonstrating the effect of CBAM and stochastic-depth regularization on class-wise precision, recall, F1-score, and accuracy for the proposed DualFusion-CBAM-Stochastic model.

Models	Class	Precision	Recall	F1-Score	Accuracy
Baseline (DualFusion + Without CBAM)	Bacterial Leaf Spot	0.90	0.90	0.90	0.91
	Downy Mildew	0.83	0.88	0.85	
	Healthy Leaf	0.95	0.98	0.96	
	Mosaic Disease	0.90	0.90	0.90	
	Powdery Mildew	0.88	0.90	0.89	
DualFusion + With (CBAM)	Bacterial Leaf Spot	0.95	0.95	0.95	0.93
	Downy Mildew	0.90	0.90	0.90	
	Healthy Leaf	0.95	0.95	0.95	
	Mosaic Disease	0.93	0.93	0.93	
	Powdery Mildew	0.88	0.90	0.89	
Proposed (CBAM + Stochastic Depth)	Bacterial Leaf Spot	0.97	0.95	0.96	0.96
	Downy Mildew	0.95	0.97	0.96	
	Healthy Leaf	0.97	0.97	0.97	
	Mosaic Disease	0.92	0.95	0.93	
	Powdery Mildew	0.97	0.95	0.96	

## 6 State of art

Recent developments in deep learning have significantly advanced plant leaf disease classification, yet comparative studies often lack both updated transformer-based approaches and practical deployment metrics. As summarized in **Table 9**, existing models include conventional CNNs such as ResNet50, attention-enhanced networks like RBNet−Self, and transformer-based models such as SEViT, covering multiple crops including pumpkin, tomato, and wheat. While these models demonstrate competitive accuracy, they often do not generalize well under heterogeneous field conditions or complex leaf textures. The proposed DualFusion-CBAM-Stochastic model surpasses prior techniques with a 96% accuracy on the Pumpkin Leaf Disease dataset, attributed to the synergistic integration of dual-backbone feature extraction, CBAM attention, and stochastic-depth regularization. This table provides a comprehensive comparison highlighting the novelty and effectiveness of the proposed architecture in terms of classification performance, robustness, and interpretability, addressing limitations of existing models and validating its potential for real-field deployment (**Table 9**). of the proposed DualFusion-CBAM-Stochastic model on the Pumpkin Leaf Disease dataset.

**Table 9:** Comparative analysis of recent state-of-the-art techniques for plant leaf disease classification, highlighting the superiority of the proposed DualFusion-CBAM-Stochastic model on the Pumpkin Leaf Disease dataset.

Ref.	Model	Crop/dataset	Accuracy (%)	Key notes
Khandaker et al., 2024	ResNet50	Pumpkin Leaf Disease	90.5	CNN baseline with explainability via Grad-CAM / Score-CAM
Hoque et al., 2025	ResNet50 / MobileNet	Tomato Leaf Disease	92.53	Residual learning in agricultural imagery
Prasad et al., 2024	Deep-Learning-Assisted Multiplex PCR	Field Crops	89.0	Hybrid molecular–DL diagnostic method
Haider et al., 2024	RBNet-Self (CNN + Self-Attention)	Wheat & Cotton (EuroSat)	83.10	Attention-enhanced CNN in multispecies setting
Li et al., 2024	YOLOv5 + Coordinate Attention + CARAFE	Leaf Disease Dataset	92.40	Real-scene detection, mobile potential
Zeng et al., 2023	SEViT (Vision Transformer + SE Attention)	Plant-Leaf Disease Dataset	81.10	Transformer-based; proof-of-concept in agriculture
	Proposed DualFusion-CBAM-Stochastic	Pumpkin Leaf Disease	96.0	Dual-backbone + CBAM + Stochastic-depth, optimized for real-field deployability

## 7 Conclusion and future work

This study proposed DualFusion-CBAM-Stochastic, an advanced deep-learning architecture for accurate and automated classification of pumpkin leaf diseases. By integrating DenseNet121 and EfficientNetB3 as a dual-backbone feature-extraction framework, the model effectively combines fine-grained texture analysis with multi-scale contextual representation. The addition of the Convolutional Block Attention Module (CBAM) enhances the discrimination of disease-relevant regions, while stochastic-depth regularization significantly improves generalization by reducing overfitting. Extensive experimentation on a balanced dataset of 2, 000 images demonstrated the model’s strong performance, achieving 96% accuracy and macro- and weighted-average F1-scores of 0.96, confirming its robustness and suitability for precision-agriculture applications. Future work will focus on improving generalization, deployment efficiency, and scalability. Expanding the dataset with real-world field images is essential to reinforce the model’s robustness and practical deployment feasibility. Field-level images inherently capture complex visual variability including inconsistent illumination, occlusion, heterogeneous backgrounds, and environmental noise that curated datasets do not fully represent. Incorporating such natural variability will facilitate the learning of more resilient and noise-tolerant features, thereby reducing domain shift and improving performance under real agricultural conditions. Collecting images using widely accessible devices, such as smartphones and DSLRs, supported by expert annotation from plant pathologists, will further enhance dataset diversity and ensure labeling reliability, enabling more accurate farmer-oriented diagnostic systems.

In addition to dataset expansion, future work will also investigate model-compression techniques to enable efficient deployment on mobile and edge-computing platforms. Among various approaches, quantization-aware training appears to be the most suitable for the proposed DualFusion-CBAM-Stochastic architecture. This is because quantization can substantially reduce model size and computational overhead while preserving accuracy, even in attention-enhanced and multi-backbone configurations. By converting floating-point operations to lower-precision formats during training, quantization-aware optimization ensures minimal performance degradation, making it highly effective for real-time, on-device disease diagnosis in resource-constrained agricultural environments.

Additionally, future research will evaluate model-compression strategies such as pruning, quantization, and knowledge distillation to enable real-time inference on mobile and edge devices. Integration with IoT platforms, UAV-based imaging, and geospatial monitoring infrastructures could facilitate continuous field surveillance and early disease detection. Incorporating environmental metadata (humidity, temperature, soil conditions) may further support predictive modeling and risk assessment. Finally, extending the methodology to additional crops and leveraging domain-adaptation and transfer-learning techniques will enhance scalability and broaden applicability across diverse agricultural contexts.

## Data Availability

The original contributions presented in the study are included in the article/supplementary material. Further inquiries can be directed to the corresponding authors.
